# Nogo-B promotes invasion and metastasis of nasopharyngeal carcinoma via RhoA-SRF-MRTFA pathway

**DOI:** 10.1038/s41419-022-04518-0

**Published:** 2022-01-24

**Authors:** Jingyi Wang, Qian Zhong, Hua Zhang, Shangxin Liu, Shibing Li, Tianliang Xia, Zhiwen Xiao, Renhui Chen, Yuchu Ye, Faya Liang, Ping Han, Xiaoming Huang

**Affiliations:** 1 Department of Otolaryngology-Head and Neck Surgery, Sun Yat-sen Memorial Hospital, Sun Yat-sen University, Guangzhou, China; 2 Guangdong Provincial Key Laboratory of Malignant Tumor Epigenetics and Gene Regulation, Guangzhou, China; 3 State Key Laboratory of Oncology in South China, Guangdong Key Laboratory of Nasopharyngeal Carcinoma Diagnosis and Therapy, Collaborative Innovation Center for Cancer Medicine, Sun Yat-sen University Cancer Center, Guangzhou, China; 4 MOE Key Laboratory of Tropical Disease Control, Centre for Infection and Immunity Studies (CIIS), Seventh Affiliated Hospital, School of Medicine, , Sun Yat-sen University, Shenzhen, Guangdong China; 5 Department of Otorhinolaryngology, Head and Neck Surgery, Department of Thyroid Center/Thyroid Surgery, The Sixth Affiliated Hospital of Sun Yat-sen University, Guangzhou, China

**Keywords:** Head and neck cancer, Drug discovery

## Abstract

Distant metastasis remains the major cause for treatment failure in patients with nasopharyngeal carcinoma (NPC). Thus, it is necessary to investigate the underlying regulation mechanisms and potential biomarkers for NPC metastasis. Nogo-B (neurite outgrowth inhibitor B), encoded by reticulon-4, has been shown to be associated with the progression and advanced stage of several cancer types. However, the relationship between Nogo-B and NPC remains unknown. In this study, we found that higher expression of Nogo-B was detected in NPC cells and tissues. Higher expression of Nogo-B was statistically relevant to N stage, M stage, and poor prognosis in NPC patients. Further functional investigations indicated that Nogo-B overexpression could increase the migration, invasion, and metastasis ability of NPC cells in vitro and in vivo. Mechanistically, Nogo-B promoted epithelial-mesenchymal transition (EMT) and enhanced the invasive potency by interacting directly with its receptor NgR3 in NPC. Additionally, overexpression of Nogo-B could upregulate the protein levels of p-RhoA, SRF, and MRTFA. A positive relationship was found between the expression of Nogo-B and the p-RhoA in NPC patients as well as in mouse lung xenografts. Nogo-B^high^ p-RhoA^high^ expression was significantly associated with N stage, M stage, and poor prognosis in NPC patients. Notably, CCG-1423, an inhibitor of the RhoA-SRF-MRTFA pathway, could reverse the invasive potency of Nogo-B and NgR3 in NPC cell lines, and decrease the expression of N-Cadherin, indicating that CCG-1423 may be a potential target drug of NPC. Taken together, our findings reveal that Nogo-B enhances the migration and invasion potency of NPC cells via EMT by binding to its receptor NgR3 to regulate the RhoA-SRF-MRTFA pathway. These findings could provide a novel insight into understanding the metastasis mechanism and targeted therapy of advanced NPC.

## Introduction

Nasopharyngeal carcinoma (NPC), a type of malignant carcinoma originating from epithelial cells, is endemic in Southeast Asia [1–3]. With improvement in the diagnosis level and the development of radiotherapy technology, the local control and overall survival rate of NPC have markedly improved; however, distant metastasis remains the main reason for treatment failure [4]. Regional lymph node metastasis occurred among ~85% of patients at the initial diagnosis and during disease progression, distant metastasis developed among an additional 15.7% of the patients [5]. Among the NPC patients with recurrence, distant metastasis might account for 40–50% within the first year and 80–97% within 5 years after treatment, respectively [6–9]. Current evaluations of the severity and therapeutic regimen of NPC patients are mainly based on TNM staging from clinical imaging examinations [10]. Recently, web-based calculators had been used to predict the personalized conditional risk of recurrence in NPC, but the evaluation system remains insufficient to accurately predict the risk of distant metastasis [11]. Therefore, the underlying mechanisms and potential biomarkers of NPC metastasis need to be identified, hopefully facilitating the prediction of metastasis and prognosis of NPC and even the development of novel therapeutic targets.

In our previous study, we had found that NgR3 was highly expressed in NPC cell lines and tissues and was associated with poor prognosis in NPC [12]. However, the mechanism of how NgR3 promotes the invasion and metastasis of NPC remains unknown. In the nervous system, NgR3 can function by binding its ligand [13]. However, whether NgR3 functions after binding its ligand in NPC remains unknown. In this study, Nogo-B (neurite outgrowth inhibitor B), a ligand of NgR3, may upregulate dramatically in NPC cell lines, rather than other ligands (Supplementary Fig. 1A). The carboxyl terminus of Nogo-B has a 188-amino acid Nogo-66 domain, which can activate GTPase/Rho and its downstream effector proteins, such as mTOR, PKC, STATs, EGFR, and other molecular pathways [14, 15]. Nogo-B is associated with the progression of gastric cancer, hepatocellular carcinoma and other carcinomas, contributing to angiogenesis, inflammation and lipid metabolism [14, 16, 17]. However, in NPC, the expression, function, and mechanisms of Nogo-B remain unknown.

In our study, we systematically demonstrated that higher expression of Nogo-B was detected in multiple NPC cell lines and was related to the poor prognosis of NPC patients. Nogo-B overexpression could increase p-RhoA expression and further enhance the migration and invasion ability of NPC cells via EMT by activating the RhoA-SRF-MRTFA pathway. Importantly, inhibition of RhoA-SRF-MRTFA pathway by CCG-1423 reversed Nogo-B-induced cancer progression, demonstrating that CCG-1423 may be a potential target drug of NPC.

## Materials and methods

### Tissues

The study included 116 patients diagnosed with NPC between January 2016 and February 2018 at Sun Yat-sen Memorial Hospital, Sun Yat-sen University. Primary tumor samples, TNM stage (UICC/AJCC 8th edition, 2017), treatment, and other demographic data were collected. The beginning of follow-up duration was the complement of NPC treatment, and overall survival (OS), progression-free survival (PFS), and disease-free survival (DFS) were all considered as endpoints. During the median follow-up of 44 months (8–61 months), one patient had local recurrence, 15 patients had distant metastasis, one patient had both local and regional recurrence, one patient had both distant metastasis and regional recurrence.

Twelve NPC tissues and nine noncancerous nasopharyngeal epithelial tissues collected between September 2018 and December 2018 at Sun Yat-sen Memorial Hospital, Sun Yat-sen University. These tissues were used to measure Nogo-B expression by real-time PCR. All the biopsy tissues for real-time PCR were immediately immersed in RNA-Later solution (R0901, Sigma, USA) overnight at 4 °C and then preserved at −80 °C.

All the patients were informed and agreed on the use of these clinical materials for research purposes. This study was approved by the Institute Research Ethics Committee of Sun Yat-sen Memorial Hospital, Sun Yat-sen University, and followed the guidelines of the Helsinki Declaration.

### Cell lines and cell cultures

Nasopharyngeal epithelial cell lines NP69(RRID: CVCL_F755) was cultured in a keratinocyte serum-free medium (KSF, Invitrogen, USA). The NPC cell lines including HK1 (RRID:CVCL_7047), TW03(RRID:CVCL_6010), SUNE1(RRID: CVCL_6946), 5–8 F(RRID:CVCL_C528), 6-10B(RRID: CVCL_C529), CNE2(RRID: CVCL_6889), HONE1(RRID: CVCL_8706) were cultured in RPMI 1640 medium (Gibco, USA) with 5% fetal bovine serum (Gibco, USA). HEK293T(CVCL_0063) cells were cultured in DMEM (Gibco, USA) with 10% fetal bovine serum (Gibco, USA). The cell lines were cultured in 37 °C with 5% CO_2_. All the cell lines in this study have been authenticated using STR profiling within the last 3 years and all experiments were performed with mycoplasma-free cells.

### Immunohistochemistry (IHC)

The procedure of IHC had been performed according to the previous reports [12]. The antibodies included in this study and the concentration were listed as follows: anti-Nogo-B (1:400, ab180847, Abcam); anti-p-RhoA (Ser188) (1:100, PA5-105763, ThermoFisher); anti-E-Cadherin (1:100, 610818, BD).

### Histological evaluation

For each slide, five fields of vision were selected randomly for scoring and the average score was used in final analysis. The immunoreactivity score (IRS) was performed according to the previous reports [12]. All results were confirmed by at least two pathology experts through a double-blind analysis. Nogo-B IRS and p-RhoA IRS are both continual variables, ROC curves were used to determine cutoff values [18–20]. When the Youden index is at its maximum (0.361), the cutoff value of Nogo-B IRS is 5(Supplementary Fig. 1B) and was used to classify the patients’ cohort into low Nogo-B expression groups (IRS < 5.0) and high Nogo-B expression groups (IRS ≥ 5.0), as well as the determination of p-RhoA IRS (Supplementary Fig. 1B).

### RNA extraction and real-time PCR analysis

Total RNA from different cell lines and human samples were extracted using TRIzol reagent (Invitrogen, Carlsbad, CA). The procedure of real-time PCR had been performed according to the previous reports [12]. The relevant primers were attached in Supplementary Table 2.

### Western blotting

Cells were lysed in lysis buffer supplemented with protease inhibitor cocktail (TargetMol, USA) at 4 °C for 20 min. The western blotting assay was performed as described previously [21].

### Coimmunoprecipitation (Co-IP)

Cells were collected using a cell scraper and suspended in a cold IP buffer containing protease inhibitor cocktail. Then, the mixtures were incubated at 4 °C with inversion for 1 h and centrifuged. Take 30 µl supernatants as input and the left supernatants were incubated with Protein A/G beads (Thermo Scientific, USA) linked to the corresponding antibodies at 4 °C with inversion overnight. The mixtures were centrifuged, and the precipitates were washed five times with cold IP buffer. The precipitates were collected for Western Blotting.

### Immunofluorescence (IF)

The procedure of IF had been performed according to the previous reports [12]. Confocal images were acquired using a confocal laser scanning microscope (OLYMPUS FV1000, Japan).

### Plasmids

The full-length human Nogo-B opening reading frame (ORF) was cloned into pcDNA3.1 + (Clontech, USA), pcDNA6/myc-His B and pLVX-DsRed-Monomer-N1(Clontech, USA). The relevant primers were attached in Supplementary Table 1.

### Establishment of stable cell lines

Cell transfection was performed using polyethyleneimine (Polyscience, China) according to the manufacturer’s instructions. The lentiviral plasmids pLVX-DsRed-Monomer-N1 were cotransfected with psPAX2 (Clontech, USA) and pMD2.G (Clontech, USA) at a mass ratio of 1:0.75:0.25 into HEK293T cells using polyethyleneimine. After 36–48 h, the viral supernatants were collected and filtered with a 0.45 mm filter and used for transduction with polybrene (107689, Sigma-Aldrich, USA). Thirty-six hours after transduction, the medium was exchanged for a fresh culture medium. The cells were cultured for 24 h, and then 1–2 μg/mL of puromycin was added to the medium according to the cell drug sensitivities to select the stably transfected cells for at least one week.

### RNA interference

For siRNA transfection, siRNA oligoribonucleotides (RiboBio Co., Ltd.) were used together with Lipofectamine RNAiMAX reagents (Life Technologies, USA) according to the manufacturer’s instructions. The siRNA sequences included in this study were as follows: NgR3 siRNA: 5′-GGATCTACTCGAACAACAT-3'; Nogo-B-siRNA: 5'-GAGCGTAACAGCCTACATT-3'.

### Transwell assay

NPC cell lines (3–5 × 10^4^ cells for the migration assay and 6–10 × 10^4^ cells for the invasion assay) were plated in serum-free RPMI 1640 medium in the upper chambers of inserts (Corning, USA) in a 24-well plate. The upper chambers of inserts were precoated with Matrigel for invasion assay. HK1, TW03, and CNE2 cells were used for the migration assay and the invasion assay as described previously [18]. All experiments were done in triplicate.

### 3-(4,5-Dimethylthiazol-2-yl)-2,5-diphenyltetrazolium bromide (MTT) assay

The cell lines were used for MTT assay as described in our previous paper [18]. Cells were seeded at 400–1000 cells/well in a 96-well plate in sextuplicate. All experiments were repeated three times.

### Lung metastasis model

The animal experiments were performed in BALB/c-nude female mice (16–18 g) at age 5–6 weeks, which were purchased from Model Organisms Laboratories (Shanghai, China). Lung metastasis models were established as described in our previous study [18]. CNE2 (5 × 10^5^) and TW03 (1 × 10^6^) cells stably overexpressing vector or Nogo-B were inoculated through the tail vein, and the nude mice were euthanized at the 6–8^th^ weeks. Then the lungs of each mouse were collected. Part of the lung was removed and stored at −80 °C, and the remaining tissues were fixed in 4% paraformaldehyde in PBS overnight and embedded in paraffin for H&E staining and IHC. All the nude mice were randomly allocated to different groups. All the animal studies were approved by the Institutional Animal Care and Use Committee of Sun Yat-sen University.

### Other reagents

The reagents used were as follows: rabbit monoclonal antibodies against p-FAK (Tyr397) (1:1000, ab81298, Abcam), flag(1:1000, 14793 S, Cell Signaling Technology), myc (1:1000, 2278t, Cell Signaling Technology), SRF (1:500, #5147, Cell Signaling Technology), N-Cadherin (1:1000, #13116, Cell Signaling Technology), NgR3 (1:1000, NBP1-92359, Novus); rabbit polyclonal antibodies against FAK (1:1000, #3285 S, Cell Signaling Technology dilution); p-SRF (Ser103) (1:500, #4261, Cell Signaling Technology); Alexa Fluor® 594 (or 488)-conjugated goat-anti-mouse (or rabbit) (1:2000, ThermoFisher); mouse monoclonal antibodies against GAPDH (1:2000, KC-5G4, Kangcheng Biotech). Nuclear dye against 40–60-diamidino-2-phenylindole (DAPI; 1:2000, Molecular Probes).

### Statistical analysis

The Statistical Package for Social Sciences, version 25.0 (SPSS, Inc., Chicago, USA), was used for the statistical analysis. The association of the Nogo-B, p-RhoA or Nogo-B^+^p-RhoA^+^ levels with NPC patients’ clinicopathological features was analyzed by either *χ*^2^-test or Fisher’s exact test. Differences among variables were assessed by 2-tailed Student’s *t*-test. Survival curves were constructed using Kaplan–Meier survival analysis and compared by the log-rank test. Univariate and multivariate regression analysis was performed using the Cox proportional hazards model to determine the significance of particular prognostic factors on survival. In any condition, a *P*-value < 0.05 was considered statistically significant.

## Results

### Expression of Nogo-B in NPC cell lines and tissues

**Fig. 1:**
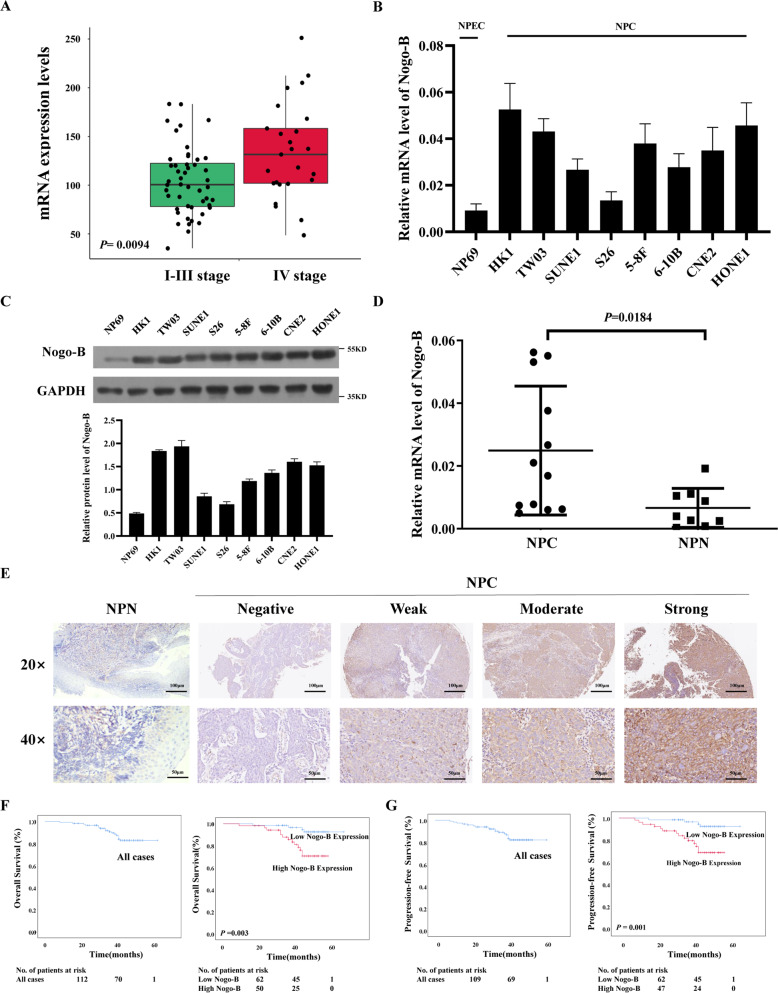
Nogo-B is overexpressed in NPC and correlates with a poor prognosis of NPC. **A** Average expression level of Nogo was highly expressed in stage IV NPC tissues in the GSE102349 dataset. **B** The mRNA expression of Nogo-B was upregulated in the NPC cell lines (HK1, TW03, SUNE1, S26, 5–8 F, CNE2, and HONE1), as demonstrated by real-time PCR. **C** Western blotting showed that the expression of Nogo-B was upregulated in the NPC cell lines (HK1, TW03, SUNE1, S26, 5–8 F, CNE2, and HONE1). **D** The expression of Nogo-B was higher in NPC tissues (NPC) than in nontumorous tissues (NPN), as demonstrated by real-time PCR (*P* = 0.0184). **E** Representative IHC results from nontumorous tissues (left) and NPC tissues with different levels of Nogo-B protein, as detected by IHC. **F** The 3-year OS rate of 116 NPC patients was 85.3%. Kaplan–Meier and log-rank test analysis showed that the cumulative 3-year OS rate was 93.7% in the low Nogo-B expression group (blue line) and 75.5% in the high Nogo-B expression group (red line) (*P* = 0.003). **G** The 3-year PFS rate of 116 NPC patients was 84.5%. Kaplan–Meier and log-rank test analysis showed that the cumulative 3-year PFS rate was 93.7% in the low Nogo-B expression group (blue line) and 73.6% in the high Nogo-B expression group (red line) (*P* = 0.001).

We analyzed Nogo expression in the GSE102349 dataset [22], which comprises 48 tissues of stage I-III NPC and 25 of stage IV NPC. Nogo was highly expressed in stage IV disease (*P* = 0.0094) (Fig. 1A). To examine the expression of different isoforms of Nogo in NPC, we performed real-time PCR in NP69 and NPC cell lines. Nogo-A and Nogo-C showed little difference in the mRNA levels between NPC cell lines and NPECs (Supplementary Fig. 1A), while higher Nogo-B mRNA expression levels were found in NPC cell lines (Fig. 1B). NPC cell lines, including HK1, TW03, SUNE1, S26, 5–8 F, 6-10B, CNE2, and HONE1, showed higher Nogo-B expression than the NPEC cell line NP69 at the protein level (Fig. 1C).

To further explore the mRNA expression of Nogo-B in NPC patients, we performed real-time PCR in 12 NPC tissues and nine noncancerous nasopharyngeal epithelial tissues. A higher average Nogo-B expression level was detected in NPC tissues than in noncancerous nasopharyngeal epithelial tissues (*P* = 0.0184; Fig. 1D). These results indicated that Nogo-B was upregulated in NPC cell lines and tissues.

### Overexpression of Nogo-B correlates with a poor prognosis in NPC patients

To explore the protein expression levels of Nogo-B in NPC tissues, we performed IHC using an antibody against Nogo-B in 116 NPC tissues (Fig. 1E). Nogo-B was mainly localized in the cytoplasm of NPC cells. We further analyzed the association between Nogo-B expression and the clinical characteristics of NPC patients.

High expression levels of Nogo-B were observed in 53 (45.69%) NPC samples. Nogo-B expression was significantly related to N stage (*P* < 0.001), M stage (*P* = 0.023), clinical stage (*P* = 0.001), survival state (*P* = 0.006), recurrence (*P* = 0.003), locoregional failure(*P* = 0.592) and distant metastasis failure(*P* = 0.002) in NPC patients (Table 1). However, no significant correlation was found between Nogo-B expression and other clinicopathological features, such as T stage (*P* > 0.05).

**Table 1:** Correlation between the expression of Nogo-B and the clinicopathologic features of NPC.

	All cases (*n* = 116)	Nogo-B expression		*P*-value
		Low (*n* = 63)	High (*n* = 53)	
*Age (years)*				0.401
<46	52	26 (41.3%)	26 (49.1%)	
≥46	64	37 (58.7%)	27 (50.9%)	
*Gender*				0.747
Female	29	15 (23.8%)	14 (26.4%)	
Male	87	48 (76.2%)	39 (73.6%)	
*T stage*				0.532
T_1-2_	54	31 (49.2%)	23 (43.4%)	
T_3-4_	62	32 (50.8%)	30 (56.6%)	
*N stage*				<0.001*
N_0-1_	47	38 (60.3%)	9 (17.0%)	
N_2-3_	69	25 (39.7%)	44 (83.0%)	
*M stage*				0.023^a*^
M_0_	108	62 (98.4%)	46 (86.8%)	
M_1_	8	1 (1.6%)	7 (13.2%)	
*TNM stage (UICC/AJCC 8th edition, 2017)*				0.001*
I–II	38	29 (46.0%)	9 (17.0%)	
III–IV	78	34 (54.0%)	44 (83.0%)	
*State*				0.006*
Survival	99	59 (93.7%)	40 (75.5%)	
Death	17	4 (6.3%)	13 (24.5%)	
*Recurrent*				0.003*
No	98	59 (93.7%)	39 (73.6%)	
Yes	18	4 (6.3%)	14 (26.4%)	
*Locoregional failure*				0.592^a^
No	113	62 (98.4%)	51 (96.2%)	
Yes	3	1 (1.6%)	2 (3.8%)	
*Distant metastasis failure*				0.002*
No	100	60 (95.2%)	40 (75.5%)	
Yes	16	3 (4.8%)	13 (24.5%)	

*p < 0.05

^a^ Fisher’s exact test

The cumulative 3-year OS, PFS, and DFS rates of the cohort of 116 NPC patients were 85.3, 84.5, and 84.5% respectively (Fig. 1F, G, Supplementary Fig. 1C). The median follow-up time for the entire patient cohort was 44 months. The cumulative 3-year OS, PFS, and DFS rates were 75.5, 73.6, and 73.6% in the high Nogo-B expression group, whereas all 93.7% in the low Nogo-B expression group, respectively (*P* < 0.01; Fig. 1F, G, Supplementary Fig. 1C). We applied multivariable Cox regression analysis and found that M stage (*P* = 0.004) and Nogo-B expression (*P* = 0.031) were independent prognostic factors of NPC (Table 2).

**Table 2:** Univariable and multivariable analyses.

Variables	HR	95% CI	*P*-value
**Univariable analysis (*n*** = **116)**			
Age	0.973	0.375–2.522	0.955
Gender	1.036	0.338–3.180	0.950
T stage	1.733	0.641–4.690	0.279
N stage	3.493	1.003–12.164	0.049^a^
M stage	7.711	2.681–22.181	<0.001^a^
Nogo-B expression	4.668	1.519–14.344	0.007^a^
**Multivariable analysis (*n*** = **116)**			
M stage	5.087	1.706–15.169	0.004^a^
Nogo-B expression	3.583	1.121–11.453	0.031^a^

^a^*P* < 0.05

These histological results indicated that Nogo-B could be related to tumor progression and poor prognosis in NPC patients.

### Nogo-B enhances NPC cell migration and invasion

**Fig. 2:**
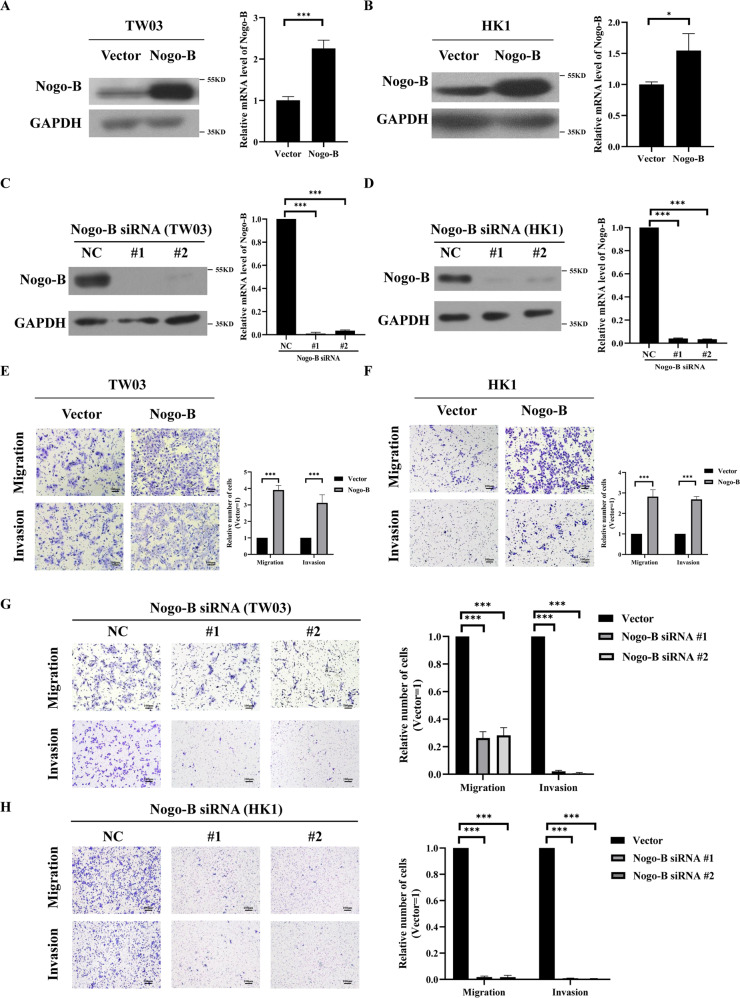
Nogo-B promotes NPC cell migration and invasion in vitro. **A** Establishment of stable cell lines (TW03) overexpressing Nogo-B, as confirmed by Western blotting and real-time PCR. **B** Establishment of stable cell lines (HK1) overexpressing Nogo-B, as confirmed by Western blotting and real-time PCR. **C** The average number of migrated and invaded cells in three fields of TW03 cell lines stably overexpressing Nogo-B increased compared with those in vector cells. **D** The average number of migrated and invaded cells in three fields of HK1 cell lines stably overexpressing Nogo-B increased compared with those in vector cells. **E** The cell lines (TW03) with Nogo-B knockdown were confirmed by Western blotting and real-time PCR. **F** The cell lines (HK1) with Nogo-B knockdown were confirmed by Western blotting and real-time PCR. **G** The average number of migrated and invaded cells in three fields of TW03 cell lines with knockdown of Nogo-B decreased compared with those in vector cells. **H** The average number of migrated and invaded cells in three fields of HK1 cell lines with knockdown of Nogo-B decreased compared with those in vector cells. Data information: *P < 0.05; **P < 0.01; ***P < 0.001; ns no significance.

To detect the function of Nogo-B in NPC cells, the NPC cell lines HK1 and TW03 were used to establish stable Nogo-B overexpressing and knockdown cell lines, respectively, a finding confirmed by real-time PCR and Western blotting (Fig. 2A–D).

To investigate the functional consequences of Nogo-B expression, invasion assays, migration assays, and MTT assays were performed in the above cell lines. Results of Transwell assays demonstrated the number of migratory or invasive cells was obviously increased in Nogo-B overexpression cell lines rather than in the control group (Fig. 2E, F, Supplementary Fig. 1D); whereas knockdown of Nogo-B by siRNA suppressed cell invasion and migration unambiguously with a statistical difference (Fig. 2G, H). Unexpectedly, Nogo-B overexpression could not influence the growth ability of HK1 and CNE2 (Supplementary Fig. 1E), suggesting that an increased number of migratory or invasive cells should not attribute to cell proliferation. Thus, these results indicated that Nogo-B overexpression could enhance the migration and invasion potency of NPC cells but could not influence the potency of cell growth.

### Nogo-B promotes lung metastasis in vivo

**Fig. 3:**
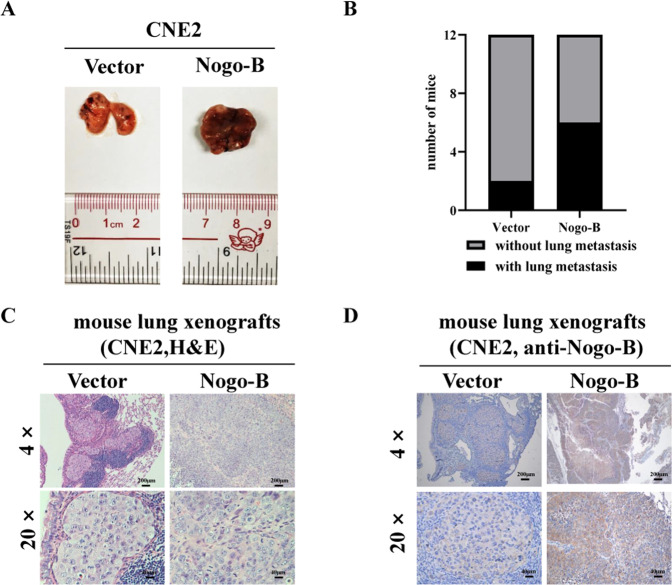
Nogo-B promotes metastasis in vivo. **A** Image of the mouse lungs in each group, respectively. **B** The ratio of developing lung metastasis of nude mice was markedly higher in the Nogo-B group than that in the vector-control group. **C** H&E staining results confirmed the existence of lung xenografts, and the size of lung xenografts of the Nogo-B group were larger in the Nogo-B group. **D** Immunohistochemical results confirmed the high expression of Nogo-B in the Nogo-B groups.

To explore whether Nogo-B promoted NPC metastasis in vivo, we constructed a lung metastasis model by injecting either CNE2 or TW03 cells stably overexpressing vector or Nogo-B. Six to eight weeks later, the mice were sacrificed, and the lungs were dissected (Fig. 3A, Supplementary Fig. 1F). Hematoxylin and eosin (H&E) staining of lung xenografts sections showed there were more nude mice with metastatic nodules in the Nogo-B overexpressing group, no matter whether parental cells were CNE2(Fig. 3B) or TW03 (Supplementary Fig. 1G). Additionally, the diameter of the metastatic lung xenografts was longer in nude mice injected with Nogo-B overexpressing cells than that in the control group (Fig. 3C, Supplementary Fig. 1H). In nude mice injected with Nogo-B overexpressing cell lines, the expression of Nogo-B was upregulated in metastatic lung xenografts (Fig. 3D). These results indicated that Nogo-B promoted NPC metastasis significantly.

### Nogo-B promotes NPC migration and invasion via its receptor NgR3

**Fig. 4:**
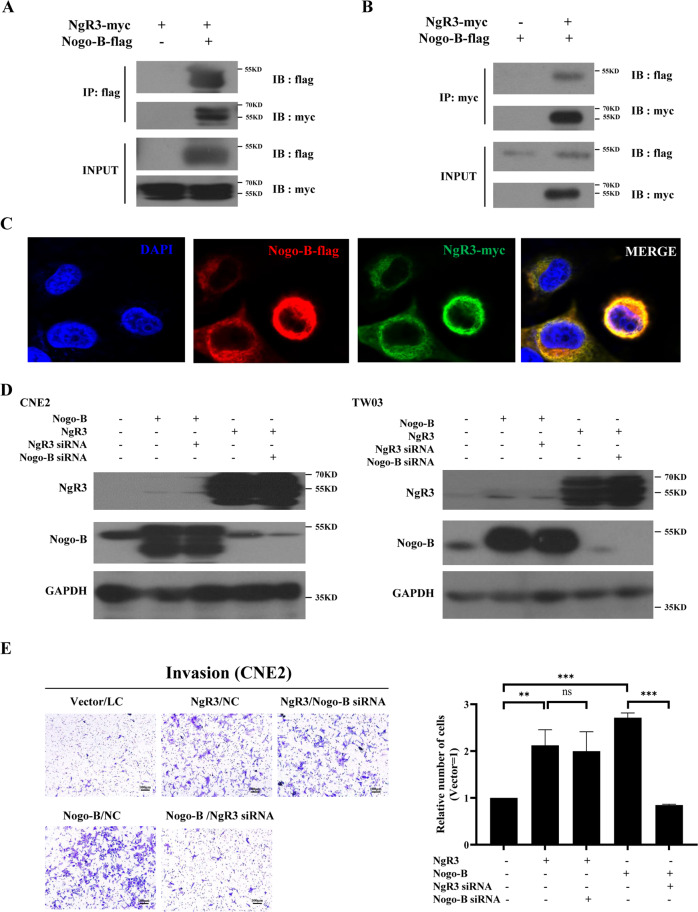
Nogo-B regulates NPC cell migration through NgR3. **A** Lysates from HEK293T cells transfected with NgR3-myc and Nogo-B-flag were subjected to immunoprecipitation using beads with anti-flag antibody, and coimmunoprecipitated NgR3-myc was detected by Western blotting. The total protein expression levels were confirmed. **B** Lysates from HEK293T cells transfected with NgR3-myc and Nogo-B-flag were subjected to immunoprecipitation using beads with anti-myc antibody, and coimmunoprecipitated Nogo-B-flag was detected by Western blotting. The total protein expression levels were confirmed. **C** CNE2 cells were transfected with NgR3-myc and Nogo-B-flag. Immunofluorescence showed that NgR3 and Nogo-B were colocalized. **D** NgR3 siRNA or Nogo-B siRNA could not influence each other’s protein expression. **E** The average number of invaded CNE2 cells in three fields decreased by NgR3 siRNA even when Nogo-B was overexpressed.

NgR3 is considered as the receptor of Nogo-B; however, the relationship between Nogo-B and NgR3 has not been shown in NPC. In this study, co-IP demonstrated that Nogo-B interacted with NgR3 directly (Fig. 4A, B). Additionally, immunofluorescence (IF) demonstrated that Nogo-B and NgR3 colocalized in the cytoplasm of NPC cells (Fig. 4C). The rescue experiments showed there was no correlation between Nogo-B and NgR3 in the protein expression levels (Fig. 4D). We investigated whether NgR3 could regulate the expression and biological function of Nogo-B in NPC. To explore the mechanism by which Nogo-B enhances NPC migration, the Transwell assay was used in NPC cell lines with stable Nogo-B or NgR3 overexpressing. The invasive ability of NPC cells was abolished by NgR3 siRNA in Nogo-B-overexpressing NPC cells (*P* < 0.001; Fig. 4E, Supplementary Fig. 1I). However, Nogo-B siRNA did not interfere with the invasive ability in NgR3-overexpressing NPC cells.

This result indicated that the invasive ability of Nogo-B may rely on NgR3 and could regulate NgR3’s functions, indicating that NgR3 might be the downstream molecule of Nogo-B in NPC.

### Nogo-B regulates the epithelial-mesenchymal transition

**Fig. 5:**
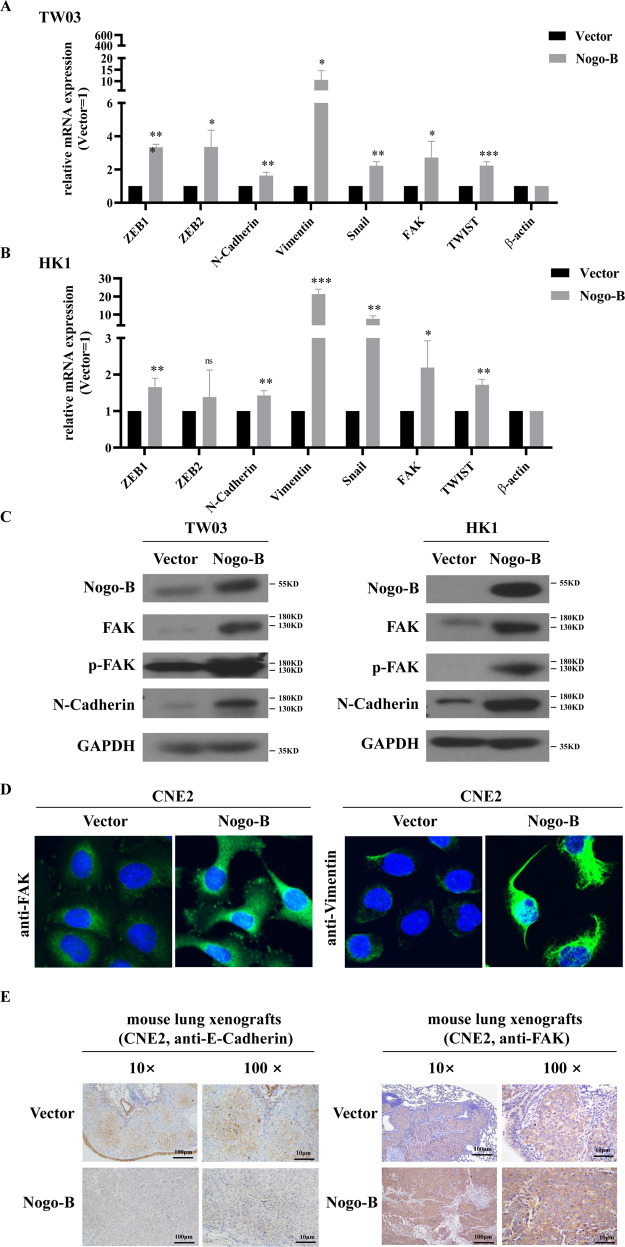
Nogo-B promotes NPC cell migration by regulating EMT. **A** Real-time PCR showed overexpressing Nogo-B increased the expression of EMT markers, such as ZEB1, ZEB2, N-Cadherin, Vimentin, Snail, FAK, TWIST, etc. in TW03 cell lines. **B** Real-time PCR showed overexpressing Nogo-B increased the expression of EMT markers, such as ZEB1, ZEB2, N-Cadherin, Vimentin, Snail, FAK, TWIST, etc. in HK1 cell lines. **C** Western blotting showed FAK, p-FAK and N-Cadherin expression increased in Nogo-B overexpressing TW03 (left) and HK1 (right) cells. **D** Immunofluorescence showed that Nogo-B upregulated the expression of FAK and Vimentin. **E** Immunohistochemistry showed that overexpressing Nogo-B downregulated E-Cadherin expression and upregulated FAK expression.

Epithelial-mesenchymal transition (EMT) is a prominent step in malignant cells [23]. In this study, overexpressing Nogo-B increased the expression of N-Cadherin, Vimentin, ZEB1 and other EMT biomarkers in the TW03 and HK1 cell lines (Fig. 5A, B), a finding that was partially confirmed by Western blotting (Fig. 5C) and IF (Fig. 5D). Furthermore, E-Cadherin decreased and FAK increased in the lung xenografts of nude mice stably overexpressing Nogo-B (Fig. 5E). These results illustrated that Nogo-B could enhance NPC cell migration and invasion by regulating the expression of EMT markers.

### Nogo-B induces NPC cell EMT through RhoA-SRF-MRTFA pathway

**Fig. 6:**
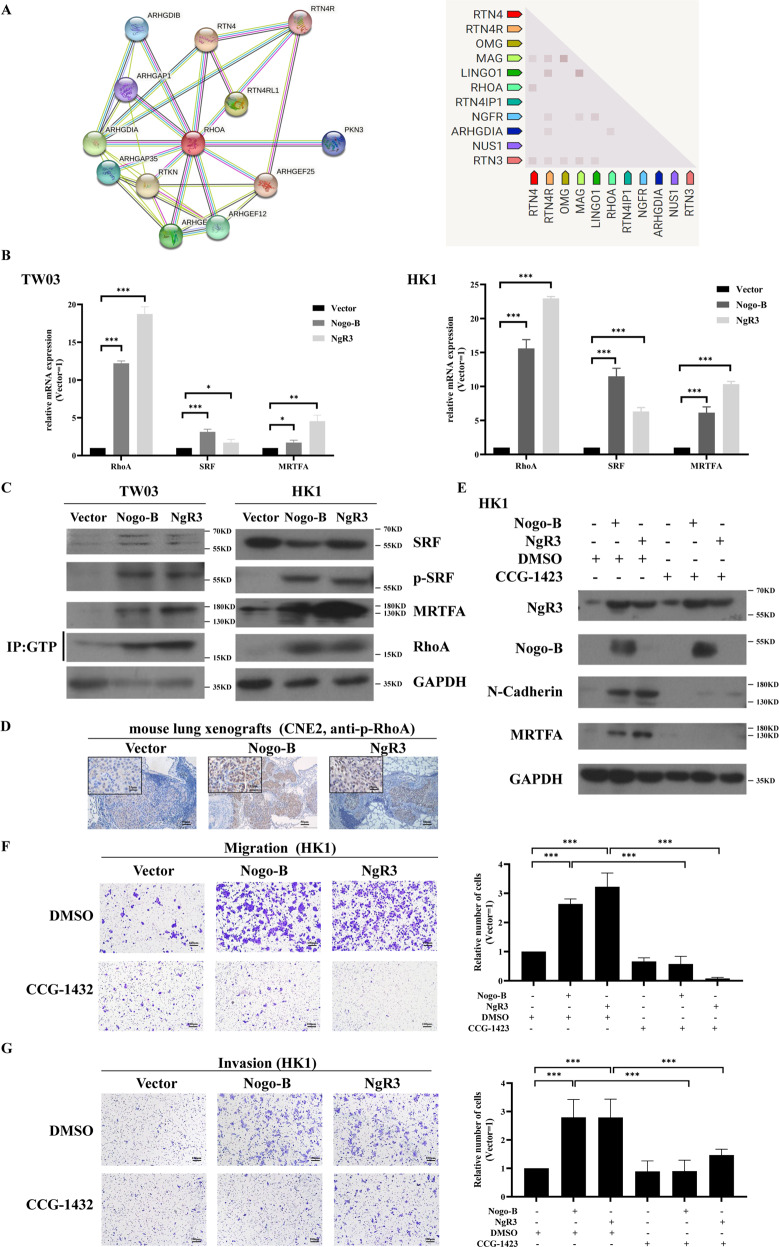
Nogo-B promotes NPC cell migration by regulating RhoA-SRF-MRTFA. **A** The STRING database showed that Nogo-B and RhoA were associated. **B** Real-time PCR showed that RhoA, MRTFA, and SRF were highly expressed in TW03 (left) and HK1 (right) cell lines overexpressing Nogo-B and NgR3. **C** Western blotting showed that RhoA-GTP, MRTFA, and SRF were highly expressed in TW03 (left) and HK1 (right) cell lines overexpressing Nogo-B and NgR3. **D** Immunohistochemistry showed that overexpressing NgR3 and Nogo-B upregulated p-RhoA expression in metastatic mouse lung xenografts. **E** After treatment with CCG-1423(30 μmol/L) for 24 h, the expression of FAK and N-Cadherin decreased in HK1 cell lines. **F** The average number of migrated cells in three fields of HK1 cell lines with overexpressing Nogo-B or NgR3 decreased after using CCG-1423(30 μmol/L). **G** The average number of invaded cells in three fields of HK1 cell lines with overexpressing Nogo-B or NgR3 decreased after using CCG-1423(30 μmol/L).

The epithelial cells undergoing EMT includes decrease of E-Cadherin, loss of cell-cell adhesion, and increase cell motility [23]. It has been reported that EMT could be regulated by RhoA GTPase in malignant carcinoma [24–26]. Thereinto, RhoA, a classical member of the Rho GTPase family, plays a fundamental role in cell adhesion [25]. In the STRING database, we identified proteins associated with Nogo and found that Nogo-B may be a potent regulator of RhoA (Fig. 6A). RhoA and its downstream genes, such as SRF and MRTFA, were significantly upregulated in the stable Nogo-B overexpressing cell lines (Fig. 6B, C). Phosphorylated RhoA (p-RhoA) expression was upregulated in the lung xenografts of nude mice stably NgR3 and Nogo-B overexpressing (Fig. 6D).

CCG-1423, an inhibitor of the RhoA-SRF-MRTFA pathway, can suppress the transcription of SRF in the RhoA signaling pathway and then blocks the activation of MRTFA [26, 27]. Using CCG-1423 [IC_50_(HK1) = 30.19 μmol/L, IC_50_(TW03) = 80.55 μmol/L; Supplementary Fig. 2A, B], the expression of N-Cadherin and MRTFA was downregulated in vitro (Fig. 6E, Supplementary Fig. 2C). Additionally, the migration and invasion abilities of Nogo-B and NgR3 could be reversed by CCG-1423 in the Transwell assay (Fig. 6F, G, Supplementary Fig. 2D, E). Therefore, p-RhoA might be a downstream biomarker of Nogo-B and further play an important role in EMT progression in NPC.

### Nogo-B is coexpressed with p-RhoA in NPC tissues

**Fig. 7:**
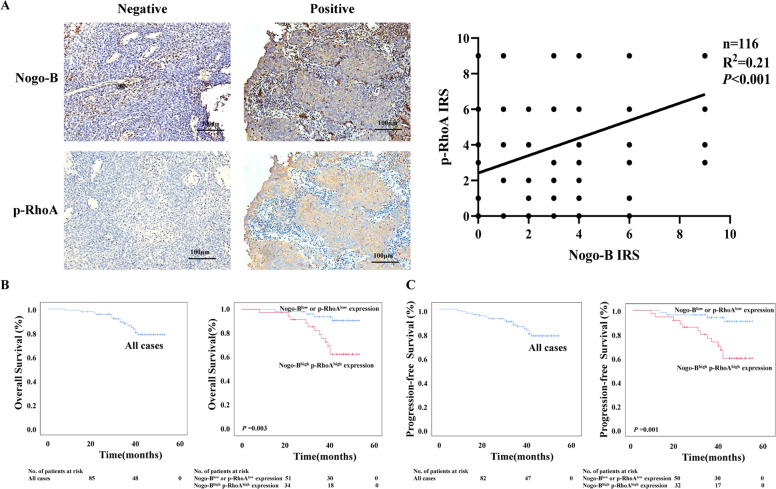
Nogo-B+ p-RhoA+ correlates with a poor prognosis of NPC. **A** Nogo-B expression was positively correlated with p-RhoA expression in NPC tissues. Left, representative images of two NPC patients were shown. Right, a positive correlation was found between Nogo-B expression and p-RhoA expression. **B** The 3-year OS rate of 89 Nogo-B^+^ p-RhoA^+^ NPC patients was 82.0%. Kaplan–Meier and log-rank test analysis showed that the cumulative 3-year OS rate was 92.5% in the low Nogo-B or p-RhoA expression group (blue line) and 66.7% in the Nogo-B^high^ p-RhoA^high^ group (red line) (*P* = 0.003). **C** The 3-year PFS rate of 89 Nogo-B^+^ p-RhoA^+^ NPC patients was 80.9%. Kaplan–Meier and log-rank test analysis showed that the cumulative 3-year PFS rate was 63.9% in the Nogo-B^high^ p-RhoA^high^ group (red line) and 92.5% in the other group (blue line) (*P* = 0.001).

We performed IHC using the antibody against p-RhoA in 116 NPC tissues. A positive relationship was found between the expression of Nogo-B and p-RhoA (*P* < 0.001) (Fig. 7A). The positive coexpression of Nogo-B and p-RhoA accounted for 89 cases (76.7%) of the NPC samples. We further analyzed the relationship between the coexpression of Nogo-B and p-RhoA (Nogo-B^+^p-RhoA^+^) and clinical characteristics in this group of NPC patients. 89 patients with Nogo-B^+^p-RhoA^+^ were divided into two groups: one comprising patients with high expression of Nogo-B and p-RhoA (Nogo-B^high^ p-RhoA^high^) and the other comprising the remaining patients (Nogo-B^low^ or p-RhoA^low^). Nogo-B^high^ p-RhoA^high^ were observed in 36 cases (40.45%). Nogo-B^high^ p-RhoA^high^ expression was significantly associated with N stage (*P* = 0.001), M stage (*P* = 0.007), clinical stage (*P* = 0.034), survival state (*P* = 0.002), recurrence (*P* = 0.001), locoregional failure (*P* = 0.563) and distant metastasis failure(*P* = 0.001) in NPC patients (Table 3).

**Table 3:** Correlation between the expression of Nogo-B^+^ p-RhoA^+^ and the clinicopathologic features of NPC.

	All cases (*n* = 89)	Nogo-B^+^ p-RhoA^+^ expression		*P*-value
		Low (*n* = 53)	High (*n* = 36)	
*Age (years)*				0.263
<46	41	27 (50.9%)	14 (38.9%)	
≥46	48	26 (49.1%)	22 (61.1%)	
*Gender*				0.960
Female	22	13 (24.5%)	9 (25.0%)	
Male	67	40 (75.5%)	27 (75.0%)	
*T stage*				0.922
T_1-2_	39	23 (43.4%)	16 (44.4%)	
T_3-4_	50	30 (56.6%)	20 (55.6%)	
*N stage*				0.001*
N_0-1_	31	26 (49.1%)	5 (13.9%)	
N_2-3_	58	27 (50.9%)	31 (86.1%)	
*M stage*				0.007^a*^
M_0_	81	52 (98.1%)	29 (80.6%)	
M_1_	8	1 (1.90%)	7 (19.4%)	
*TNM stage (UICC/AJCC 8th edition, 2017)*				0.034*
I-II	23	18 (34.0%)	5 (13.9%)	
III-IV	66	35 (66.0%)	31 (86.1%)	
*State*				0.002*
Survival	73	49 (92.5%)	24 (66.7%)	
Death	16	4 (7.5%)	12 (33.3%)	
*Recurrent*				0.001*
No	72	49 (92.5%)	23 (63.9%)	
Yes	17	4 (7.5%)	13 (36.1%)	
*Locoregional failure*				0.563^a^
No	86	52 (98.1%)	34 (94.4%)	
Yes	3	1 (1.9%)	2 (5.6%)	
*Distant metastasis failure*				0.001*
No	74	50 (94.3%)	24 (66.7%)	
Yes	15	3 (5.7%)	12 (33.3%)	

^*^p < 0.05

^a^ Fisher’s exact test

The cumulative 3-year OS, PFS, and DFS rate of the cohort of 89 Nogo-B^+^p-RhoA^+^ NPC patients were 82.0, 80.9, and 80.9% respectively (Fig. 7B, Supplementary Fig. 2F). The cumulative 3-year OS, PFS, and DFS rates were 66.7, 63.9, and 63.9% in the Nogo-B^high^ p-RhoA^high^ group, while the cumulative 3-year OS, PFS, and DFS rate were all 92.5% in the other group (*P* < 0.01, Fig. 7B, Supplementary Fig. 2F). We applied multivariable Cox regression analysis and found that M stage (*P* = 0.021) and Nogo-B^high^ p-RhoA^high^ expression (*P* = 0.036) could be independent prognostic factors of NPC (Table 4).

**Table 4:** Univariable and multivariable analyses.

Variables	HR	95% CI	*P-value*
**Univariable analysis (*n*** = **89)**			
Age	1.166	0.434–3.130	0.761
Gender	0.915	0.295–2.839	0.878
T stage	1.425	0.518–3.923	0.493
N stage	2.493	0.710–8.753	0.154
M stage	5.979	2.051–17.427	0.001^a^
Nogo-B^+^p-RhoA^+^ expression	4.669	1.506–14.481	0.008^a^
**Multivariable analysis (*n*** = **89)**			
M stage	3.751	1.223–11.505	0.021^a^
Nogo-B^+^p-RhoA^+^ expression	3.542	1.085–11.563	0.036^a^

^a^*P* < 0.05

Taken together, the above results demonstrated that Nogo-B promoted migration, invasion, and metastasis via phosphorylation of RhoA and Nogo-B^high^ p-RhoA^high^ indicates a poor prognosis in NPC patients.

## Discussion

RTN4 gene, also known as neurite outgrowth inhibitor (Nogo), produces three main isoforms (Nogo-A, Nogo-B, and Nogo-C) [15]. Nogo-A, the longest Nogo isoform, is enriched in the central nervous system and is highly expressed in brain tumors; Nogo-C is rarely reported in tumors [25, 28, 29]. However, Nogo-B shows multiple and attractive functions in different organs and tissues and is associated with many malignant cancers, such as hepatocellular carcinoma and colorectal cancer [16, 30]. Additionally, patients with high expression of Nogo-B had worse outcomes in the above cancers [12]. However, little is known about the role of Nogo-B in NPC. In our study, we found that Nogo-B expression was upregulated in NPC cell lines, but Nogo-A and Nogo-C showed no difference in the mRNA levels between NPC cell lines and NPECs, indicating the important role of Nogo-B in NPC.

We systematically explored the expression of Nogo-B in NPC. As a result, the expression of Nogo-B was upregulated in most NPC tissues and cell lines. Furthermore, the cumulative 3-year OS, PFS, and DFS rates were decreased in NPC patients with high expression of Nogo-B. Currently, the main guidance for treatment and predicting prognosis of NPC is on the basis of TNM stage system, which is insufficient to evaluate the OS, PFS, and DFS. Notably, Nogo-B may be a prognostic marker of NPC complementary to the TNM stage system. Though expression of Nogo-B was not related to T stage of patients, high expression of Nogo-B was mainly associated with N stage and M stage in NPC patients, suggesting that Nogo-B should play a more crucial role in invasion and distant metastasis than that in tumorigenesis of NPC. Therefore, in our study, we focused on the role of Nogo-B on the migration, invasion, and metastasis in NPC.

To further investigate whether Nogo-B influenced the biological function of NPC cell lines, we established cell lines with overexpression and knockdown of Nogo-B. Nogo-B could not increase proliferative potency in vitro but could promote the migration, invasion, and metastasis of NPC cells in vivo and in vitro, implying that Nogo-B may play the role of significant oncogenes in NPC. In the nervous system, Nogo-B regulates the cytoskeleton of nerve cells and influences synaptic transmission by binding with its receptor NgR3 [13, 31]. Our previous study found that NgR3 was associated with tumor progression and promoted EMT process in NPC [12]. However, the rare report has investigated the relationship between Nogo-B and NgR3 in cancers. In the present study, Nogo-B and NgR3 could colocalize and interact directly in the cytoplasm of NPC cells. Moreover, NgR3 siRNA can counteract Nogo-B-induced cell invasion in vitro. Our results demonstrated that NgR3 may act as a downstream factor of Nogo-B in NPC and then promote the migration, invasion, and metastasis of NPC in vitro.

EMT is an important biological process involved in cancer progression and development [23]. Nogo-B is reported to be associated mainly with angiogenesis, vascular remodeling, inflammation, and lipid metabolism [14, 16, 17], but the relationship between Nogo-B and EMT has not been reported in cancers. We found drastic morphological alterations and upregulation of EMT biomarkers when NPC cells were overexpressed with Nogo-B. We identified a novel function of Nogo-B in NPC by triggering the EMT process. We further investigated the underlying mechanism by which Nogo-B regulated EMT. It has been reported that RhoA and Rac1 signaling pathways promote EMT in malignant tumors [25]. The phosphorylation of RhoA can promote the cancer process and cancer cell invasion and migration in NPC [32, 33]. In our study, we found that in NPC patients, Nogo-B expression was positively correlated with p-RhoA expression, and Nogo-B^high^ p-RhoA^high^ was correlated with a poor prognosis of NPC patients, indicating a novel finding that combination of Nogo-B and p-RhoA could develop a more predictive and valuable factor for the prognosis of NPC individuals. We have learned that RhoA is an upstream regulator of SRF-MRTFA, a potential therapeutic target for malignant tumors [26], and further activates the expression of G-actin, F-actin, and other transcription factors in cancer cells [34]. SRF/MRTF induces a mesenchymal phenotypic switch in human cutaneous melanoma, and this signaling pathway axis may be a therapeutic target for malignant tumors [26]. SRF also regulates the EMT in hyperuricemic nephropathy [35]. In this study, we initially found higher expression of RhoA, SRF, and MRTFA in Nogo-B overexpressing NPC cells, indicating that Nogo-B could activate the RhoA-SRF-MRTFA pathway in vitro. Therefore, Nogo-B and RhoA likely activate RhoA-dependent SRF signaling, which in turn induces EMT progression. Therefore, Nogo-B could enhance the migration and invasion potency of NPC cells, importantly, by binding directly to its receptor NgR3 and then regulating RhoA-SRF-MRTFA pathway.

CCG-1423 is a blocker of the RhoA-SRF-MRTFA pathway and can selectively inhibit SRF-mediated transcription activated by RhoA signaling [26, 27, 36]. In this study, we newly found that CCG-1423 could affect the expression level of EMT biomarkers and inhibit the migration and invasion ability prompted by Nogo-B or NgR3 in NPC cells. This finding illustrated that SRF transcription may be important in Nogo-B signaling pathway and CCG-1423 may be a potential therapeutic target drug for NPC patients with high Nogo-B expression. In our future study, we will further explore the efficiency and toxicology of CCG-1423, promoting the development of personalized, targeted therapies for NPC patients. Additionally, the underlying mechanisms are unclear, including how Nogo-B triggers the phosphorylation of RhoA and EMT progression.

In summary, we found Nogo-B may be a valuable biomarker for NPC prognosis. Nogo-B could enhance the migration and invasion potency of NPC cells via EMT by binding to its receptor NgR3 and in turn activating the RhoA-SRF-MRTFA pathway. It implies that CCG-1423 may be a potential target drug for Nogo-B overexpressing NPC patients.

## Supplementary information

Supplement Figure Legends

Supplement Table 1

Supplement Table 2

Supplementary Figure 1

Supplementary Figure 2

confirmation of author changes

## Data Availability

The data that support the findings of this study are available from the corresponding author upon reasonable request. The data that support the findings of this study are available from the corresponding author upon reasonable request.

## References

[1] Chen YP, Chan ATC, Le QT, Blanchard P, Sun Y, Ma J (2019). Nasopharyngeal carcinoma. Lancet..

[2] Lydiatt WM, Patel SG, O’Sullivan B, Brandwein MS, Ridge JA, Migliacci JC (2017). Head and neck cancers-major changes in the American joint committee on cancer eighth edition cancer staging manual. CA Cancer J Clin.

[3] Bray F, Ferlay J, Soerjomataram I, Siegel RL, Torre LA, Jemal A (2018). Global cancer statistics 2018: GLOBOCAN estimates of incidence and mortality worldwide for 36 cancers in 185 countries. CA Cancer J Clin.

[4] Colevas AD, Yom SS, Pfister DG, Spencer S, Adelstein D, Adkins D (2018). NCCN Guidelines Insights: head and neck cancers, version 1.2018. J Natl Compr Canc Netw.

[5] Ho FC, Tham IW, Earnest A, Lee KM, Lu JJ (2012). Patterns of regional lymph node metastasis of nasopharyngeal carcinoma: a meta-analysis of clinical evidence. BMC Cancer.

[6] Chen YP, Liu X, Zhou Q, Yang KY, Jin F, Zhu XD (2021). Metronomic capecitabine as adjuvant therapy in locoregionally advanced nasopharyngeal carcinoma: a multicentre, open-label, parallel-group, randomised, controlled, phase 3 trial. Lancet.

[7] Wu LR, Zhang XM, Xie XD, Lu Y, Wu JF, He X (2019). Validation of the 8th edition of AJCC/UICC staging system for nasopharyngeal carcinoma: results from a non-endemic cohort with 10-year follow-up. Oral Oncol.

[8] Zhang Y, Chen L, Hu GQ, Zhang N, Zhu XD, Yang KY (2019). Gemcitabine and cisplatin induction chemotherapy in nasopharyngeal carcinoma. N. Engl J Med.

[9] Sun X, Su S, Chen C, Han F, Zhao C, Xiao W (2014). Long-term outcomes of intensity-modulated radiotherapy for 868 patients with nasopharyngeal carcinoma: an analysis of survival and treatment toxicities. Radiother Oncol.

[10] Qiang M, Li C, Sun Y, Sun Y, Ke L, Xie C (2021). A prognostic predictive system based on deep learning for locoregionally advanced nasopharyngeal carcinoma. J Natl Cancer Inst.

[11] Wu CF, Lv JW, Lin L, Mao YP, Deng B, Zheng WH (2021). Development and validation of a web-based calculator to predict individualized conditional risk of site-specific recurrence in nasopharyngeal carcinoma: analysis of 10,058 endemic cases. Cancer Commun (Lond).

[12] He JY, Han P, Zhang Y, Liu YD, Song SJ, Feng GK (2018). Overexpression of Nogo receptor 3 (NgR3) correlates with poor prognosis and contributes to the migration of epithelial cells of nasopharyngeal carcinoma patients. J Mol Med (Berl).

[13] Dickendesher TL, Baldwin KT, Mironova YA, Koriyama Y, Raiker SJ, Askew KL (2012). NgR1 and NgR3 are receptors for chondroitin sulfate proteoglycans. Nat Neurosci.

[14] Pathak GP, Shah R, Kennedy BE, Murphy JP, Clements D, Konda P (2018). RTN4 knockdown dysregulates the AKT pathway, destabilizes the cytoskeleton, and enhances paclitaxel-induced cytotoxicity in cancers. Mol Ther.

[15] Schwab ME (2010). Functions of Nogo proteins and their receptors in the nervous system. Nat Rev Neurosci.

[16] Tian Y, Yang B, Qiu W, Hao Y, Zhang Z, Yang B (2019). ER-residential Nogo-B accelerates NAFLD-associated HCC mediated by metabolic reprogramming of oxLDL lipophagy. Nat Commun.

[17] Cai H, Saiyin H, Liu X, Han D, Ji G, Qin B (2018). Nogo-B promotes tumor angiogenesis and provides a potential therapeutic target in hepatocellular carcinoma. Mol Oncol.

[18] Li SB, Liu YY, Yuan L, Ji MF, Zhang A, Li HY (2020). Autocrine INSL5 promotes tumor progression and glycolysis via activation of STAT5 signaling. EMBO Mol Med.

[19] Colak E, Mutlu F, Bal C, Oner S, Ozdamar K, Gok B (2012). Comparison of semiparametric, parametric, and nonparametric ROC analysis for continuous diagnostic tests using a simulation study and acute coronary syndrome data. Comput Math Methods Med.

[20] Wang HY, Sun BY, Zhu ZH, Chang ET, TO KF, Hwang JS (2011). Eight-signature classifier for prediction of nasopharyngeal [corrected] carcinoma survival. J Clin Oncol.

[21] Han P, Chen RH, Wang F, Zeng JY, Yu ST, Xu LH (2017). Novel chimeric transcript RRM2-c2orf48 promotes metastasis in nasopharyngeal carcinoma. Cell Death Dis.

[22] Zhang L, MacIsaac KD, Zhou T, Huang PY, Xin C, Dobson JR (2017). Genomic analysis of nasopharyngeal carcinoma reveals TME-based subtypes. Mol Cancer Res.

[23] Lambert AW, Weinberg RA (2021). Linking EMT programmes to normal and neoplastic epithelial stem cells. Nat Rev Cancer.

[24] Qin CD, Ma DN, Zhang SZ, Zhang N, Ren ZG, Zhu XD (2018). The Rho GTPase Rnd1 inhibits epithelial–mesenchymal transition in hepatocellular carcinoma and is a favorable anti-metastasis target. Cell Death Dis.

[25] Ma H, Li T, Tao Z, Hai L, Tong L, Yi L (2019). NKCC1 promotes EMT-like process in GBM via RhoA and Rac1 signaling pathways. J Cell Physiol.

[26] Lionarons DA, Hancock DC, Rana S, East P, Moore C, Murillo MM (2019). RAC1(P29S) Induces a mesenchymal phenotypic switch via serum response factor to promote melanoma development and therapy resistance. Cancer Cell.

[27] Sato T, Verma S, Andrade CDC, Omeara M, Campbell N, Wang JS (2020). A FAK/HDAC5 signaling axis controls osteocyte mechanotransduction. Nat Commun.

[28] Zhao X, Wang X, You Y, Wen D, Feng Z, Zhou Y (2020). Nogo-B fosters HCC progression by enhancing Yap/Taz-mediated tumor-associated macrophages M2 polarization. Exp Cell Res.

[29] Jin SG, Ryu HH, Li SY, Li CH, Lim SH, Jang WY (2016). Nogo-A inhibits the migration and invasion of human malignant glioma U87MG cells. Oncol Rep..

[30] Kawaguchi N, Tashiro K, Taniguchi K, Kawai M, Tanaka K, Okuda J (2018). Nogo-B (Reticulon-4B) functions as a negative regulator of the apoptotic pathway through the interaction with c-FLIP in colorectal cancer cells. Biochim Biophys Acta Mol Basis Dis.

[31] Wills ZP, Mandel-Brehm C, Mardinly AR, McCord AE, Giger RJ, Greenberg ME (2012). The nogo receptor family restricts synapse number in the developing hippocampus. Neuron.

[32] Fan C, Qu H, Xiong F, Tang Y, Tang T, Zhang L (2021). CircARHGAP12 promotes nasopharyngeal carcinoma migration and invasion via ezrin-mediated cytoskeletal remodeling. Cancer Lett.

[33] Yuan J, Chen L, Xiao J, Qi XK, Zhang J, Li X (2019). SHROOM2 inhibits tumor metastasis through RhoA-ROCK pathway-dependent and -independent mechanisms in nasopharyngeal carcinoma. Cell Death Dis.

[34] Kim JG, Islam R, Cho JY, Jeong H, Cap KC, Park Y (2018). Regulation of RhoA GTPase and various transcription factors in the RhoA pathway. J Cell Physiol.

[35] Zhao L, Li C, Zhou B, Luo C, Wang Y, Che L (2019). Crucial role of serum response factor in renal tubular epithelial cell epithelial–mesenchymal transition in hyperuricemic nephropathy. Aging (Albany NY).

[36] Wu T, Wang H, Xin X, Yang J, Hou Y, Fang M (2020). An MRTF-A-Sp1-PDE5 axis mediates angiotensin-II-induced cardiomyocyte hypertrophy. Front Cell Dev Biol.

