# Supplementary material for: Nogo-B promotes invasion and metastasis of nasopharyngeal carcinoma via RhoA-SRF-MRTFA pathway
Source: Cell Death Dis. 2022 Jan 24;13(1):76. doi: 10.1038/s41419-022-04518-0 (PMC8786944; doi:10.1038/s41419-022-04518-0)
Supplement: Supplementary file 1 — Supplement Figure Legends [file 41419_2022_4518_MOESM1_ESM.docx]

**Supplement Figure Legends**

**Supplementary Figure 1. Nogo-B promotes NPC cell migration.**

1. Real-time PCR showed that there was little difference in the expression of MAG, Nogo-A and Nogo-C between NPC cell lines and NPECs.
2. The ROC curve of Nogo-B and p-RhoA in the whole cohort was shown.
3. The three-year DFS rate of 116 NPC patients was 84.5%. Kaplan–Meier and log-rank test analysis showed that the cumulative three-year DFS rate was 93.7% in the low Nogo-B expression group (blue line) and 73.6% in the high Nogo-B expression group (red line) (*P*=0.002).
4. The average number of migrated and invaded cells in three fields of CNE2 cell lines stably overexpressing Nogo-B increased compared with those in vector cells.
5. Proliferation curves of the vector and overexpressing Nogo-B cell lines (up: HK1, down: CNE2). No significant difference was observed.
6. Image of the mouse lungs in each group, respectively.
7. The ratio of developing lung metastasis of nude mice was markedly higher in the Nogo-B group than that in the vector-control group.
8. H&E staining results confirmed the existence of lung xenografts and the lung metastases of Nogo-B group was larger.
9. The average number of invaded cells in three fields of TW03 cell lines decreased by NgR3 siRNA even when Nogo-B was overexpressed.

**Supplementary Figure 2. Nogo-B promotes NPC cell migration via RhoA-SRF-MRTFA.**

1. MTT assay showed IC50 of CCG-1423 in HK1 was 30.19μmol/L.
2. MTT assay showed IC_50_ of CCG-1423 in TW03 was 80.55μmol/L.
3. CCG-1423 decreased the migration of overexpressing Nogo-B or NgR3 TW03 cells.
4. CCG-1423 decreased the invasion of overexpressing Nogo-B or NgR3 TW03 cells.
5. After treatment with CCG-1423(57 μmol/L) for 24 hours, the expression of FAK and N-Cadherin decreased in CNE2 cell lines.
6. The three-year DFS rate of 89 Nogo-B^+^ p-RhoA^+^ NPC patients was 80.9%. Kaplan–Meier and log-rank test analysis showed that the cumulative three-year DFS rate was 92.5% in the low Nogo-B or p-RhoA expression group (blue line) and 63.9% in the Nogo-B^high^ p-RhoA^high^ group (red line) (*P*=0.001).
