# Supplementary material for: Nogo-B promotes invasion and metastasis of nasopharyngeal carcinoma via RhoA-SRF-MRTFA pathway
Source: Cell Death Dis. 2022 Jan 24;13(1):76. doi: 10.1038/s41419-022-04518-0 (PMC8786944; doi:10.1038/s41419-022-04518-0)
Supplement: Supplementary file 2 — Supplement Table 1 [file 41419_2022_4518_MOESM2_ESM.docx]

**Supplementary Table 1. Primer sequences for plasmid**

| **Plasmid** | **Sense (5’-3’)**  **(Restriction Enzyme sites)** | **Antisense (5’-3’)**  **(Restriction Enzyme sites)** |
| --- | --- | --- |
| **pcDNA3.1** | (BamH I) ACCGAGCTCGGATCCATGGAAGACCTGGACCAGTCTCCT | (EcoR I) GATATCTGCAGAATTCCATCACTTATCGTCGTCATCCTTGTAATCTTCAGCTTTGCGCTTCAATCCAG |
| **pcDNA6 myc-His B** | (BamH I)  ACCGAGCTCGGATCCATGGAAGACCTGGACCAGTCTCCT | (EcoR I)  GATATCTGCAGAATTCCATTCAGCTTTGCGCTTCAATCCA |
| **pLVX-DsRed-Monomer-N1** | (EcoR I)  TCGAGCTCAAGCTTCGAATTC ATGGAAGACCTGGACCAGTCTCCTC | (BamH I)  ATGGTGGCGACCGGTGGATCCTCACTTATCGTCGTCATCCTTGTAATCTTCAGCTTTGCGCTTCAATCCAG |
