# Supplementary material for: Nogo-B promotes invasion and metastasis of nasopharyngeal carcinoma via RhoA-SRF-MRTFA pathway
Source: Cell Death Dis. 2022 Jan 24;13(1):76. doi: 10.1038/s41419-022-04518-0 (PMC8786944; doi:10.1038/s41419-022-04518-0)
Supplement: Supplementary file 3 — Supplement Table 2 [file 41419_2022_4518_MOESM3_ESM.docx]

**Supplementary Table 2. Primer sequences for real-time PCR**

| **Target** | **Sense (5’-3’)** | **Antisense (5’-3’)** |
| --- | --- | --- |
| **Nogo-B** | GCTCCTCGGGCTCAGTGGTTGTTGAC | TGGCCTTCATCTGATTTCTGGATAGC |
| **ZEB1** | CCGAGCCTCCAACTTTACCT | AACCTTGTTGCTAGGGACCG |
| **ZEB2** | ACGGTATTGCCAACCCTCTG | GGTCTGGATCGTGGCTTCTG |
| **N-Cadherin** | GCGTCTGTAGAGGCTTCTGG | TGGAAAGCTTCTCACGGCAT |
| **Vimentin** | CGTATGGCGCCTCTCCAAAG | GCTAAAGCCTGTCTTTGCTCG |
| **Snail** | CCAGTGCCTCGACCACTATG | TGCAGCTCGCTGTAGTTAGG |
| **FAK** | TCGAGGCAGTATTGACAGGG | AGGATTTCTTTCCGCCCAATTC |
| **TWIST** | GGACAGTGATTCCCAGACGG | CCTTTCAGTGGCTGATTGGC |
| **RhoA** | GTCCACGGTCTGGTCTTCAG | CAGCCATTGCTCAGGCAAC |
| **MRTFA** | TGCACTGAGAAGGGGATATTCT | GATCCCTTGGCTCACCAGTT |
| **SRF** | CTTGCTGAGTGAAGGGGCCTAT | GATCATGGGCTGCAGTTTTCG |
| **β-actin** | AGTCATTCCAAATATGAGATGCGTT | TGTGGACTTGGGAGAGGACT |
