# Supplementary figures and images for: Nogo-B promotes invasion and metastasis of nasopharyngeal carcinoma via RhoA-SRF-MRTFA pathway
Source: Cell Death Dis. 2022 Jan 24;13(1):76. doi: 10.1038/s41419-022-04518-0 (PMC8786944; doi:10.1038/s41419-022-04518-0)

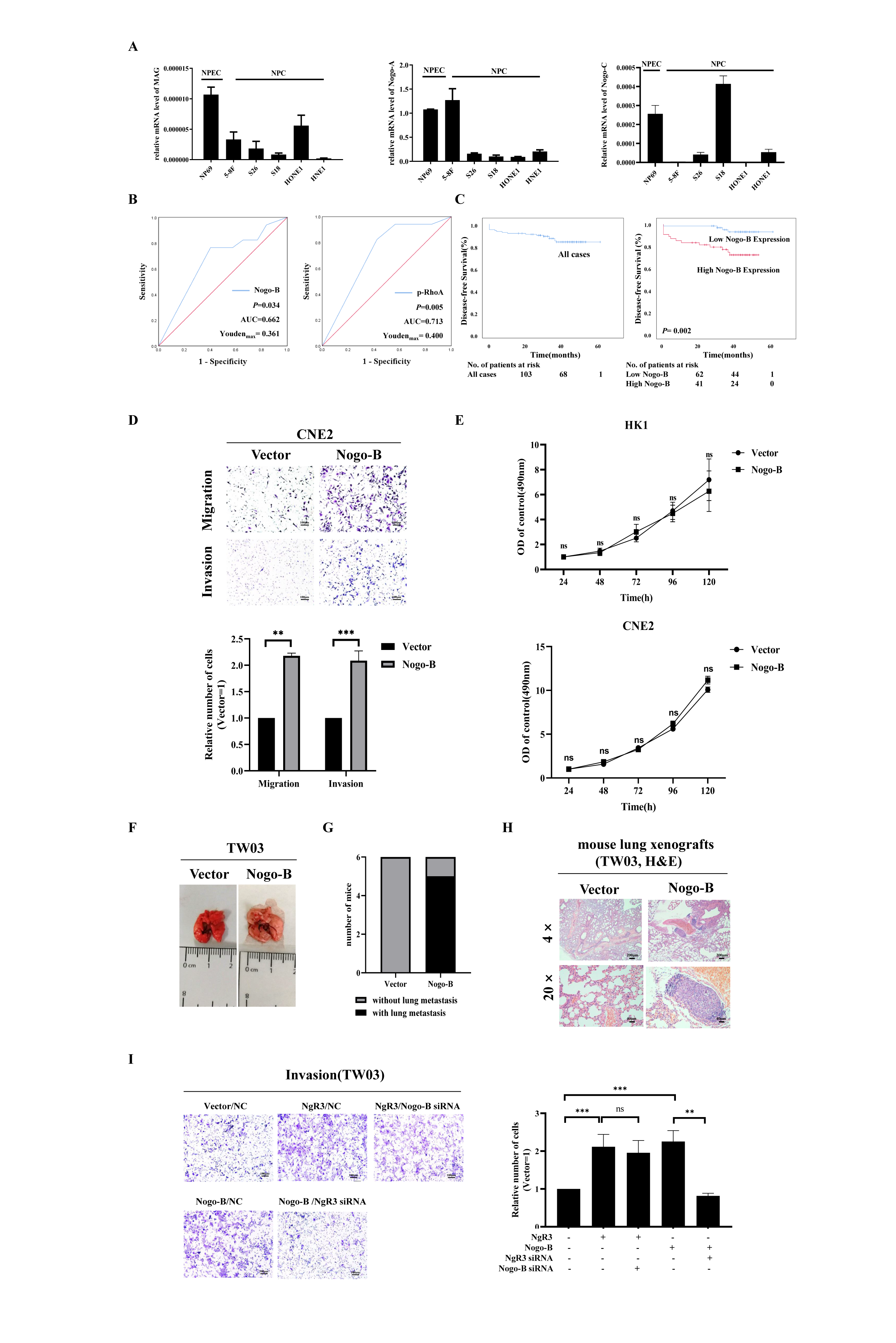

Supplement: Supplementary file 4 — Supplementary Figure 1 [file 41419_2022_4518_MOESM4_ESM.tif]

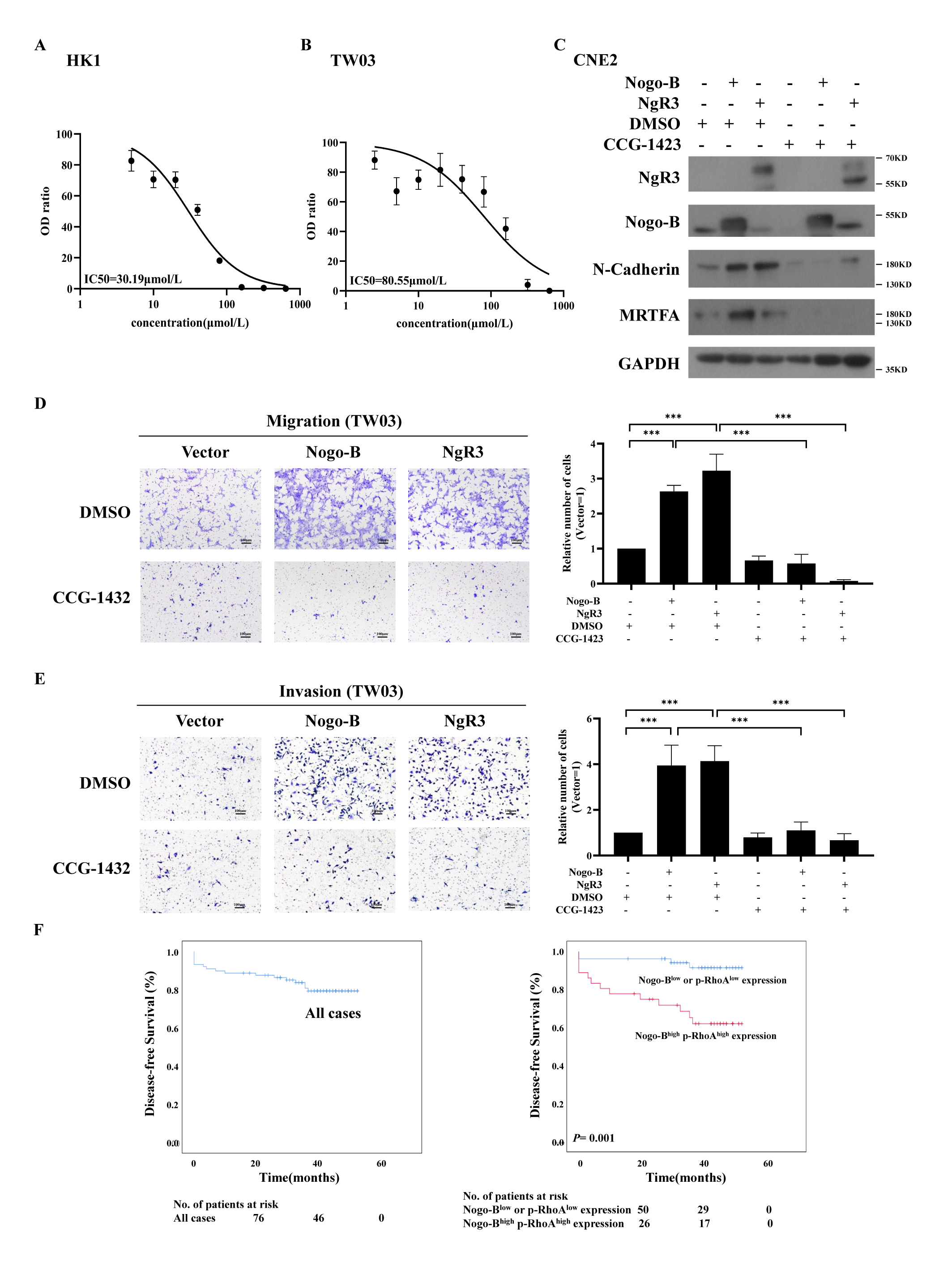

Supplement: Supplementary file 5 — Supplementary Figure 2 [file 41419_2022_4518_MOESM5_ESM.tif]
