# Supplementary material for: Nogo-B promotes invasion and metastasis of nasopharyngeal carcinoma via RhoA-SRF-MRTFA pathway
Source: Cell Death Dis. 2022 Jan 24;13(1):76. doi: 10.1038/s41419-022-04518-0 (PMC8786944; doi:10.1038/s41419-022-04518-0)
Supplement: Supplementary file 6 — confirmation of author changes [file 41419_2022_4518_MOESM6_ESM.pdf]

回复:CDDIS-21-3186RR Author confirmation email

发件人： "王静怡" <wangjyi2@mail2.sysu.edu.cn>

收件人： "黄晓明" <hxming@mail.sysu.edu.cn>

2022-01-06 21:04:10

Dear all,

I agree to the changes.

Yours sincerely,

Jingyi Wang

---------------原始邮件---------------
发件人："黄晓明 "<hxming@mail.sysu.edu.cn>;
发送时间：2022年1月6日(星期四) 下午5:23
收件人："hxming" <hxming@mail.sysu.edu.cn>;"王静怡" <wangjyi2@mail2.sysu.edu.cn>;"zhongqian" <zhongqian@sysucc.org.cn>;"zhangh255" <zhangh255@mail.sysu.edu.cn>;"liusx" <liusx@sysucc.org.cn>;"lishb" <lishb@sysucc.org.cn>;"xiatl" <xiatl@sysucc.org.cn>;"xiaozhw3" <xiaozhw3@mail.sysu.edu.cn>;"chenrh23" <chenrh23@mail.sysu.edu.cn>;"yeych6" <yeych6@mail2.sysu.edu.cn>;"liangfy3" <liangfy3@mail.sysu.edu.cn>;"hanping5" <hanping5@mail.sysu.edu.cn>;
主题：CDDIS-21-3186RR Author confirmation email

Dear co-authors,

According to the contribution work, the co-authors of our manuscript entitled "Nogo-B Promotes Invasion and Metastasis of Nasopharyngeal Carcinoma via RhoA-SRF-MRTFA Pathway" (CDDIS-21-3186R) have been changed as follows:

Jingyi Wang[1†], Qian Zhong[2†], Hua Zhang[3], Shangxin Liu[2], Shibing Li[2], Tianliang Xia[2], Zhiwen Xiao[1,4], Renhui Chen[1], Yuchu Ye[1], Faya Liang[1], Ping Han[1*], Xiaoming Huang[1*]

1 Department of Otolaryngology-Head and Neck Surgery, Sun Yat-sen Memorial Hospital, Sun Yat-sen University, Guangzhou, China; Guangdong Provincial Key Laboratory of Malignant Tumor Epigenetics and Gene Regulation, Guangzhou, China

2 State Key Laboratory of Oncology in South China, Guangdong Key Laboratory of Nasopharyngeal Carcinoma Diagnosis and Therapy, Collaborative Innovation Center for Cancer Medicine, Sun Yat-sen University Cancer Center, Guangzhou, China

3 MOE Key Laboratory of Tropical Disease Control, Centre for Infection and Immunity Studies (CIIS), Seventh Affiliated Hospital, School of Medicine, Sun Yat-sen University, Shenzhen, Guangdong, China

4 Department of Otorhinolaryngology Head and Neck Surgery, Department of Thyroid Center/Thyroid Surgery, The Sixth Affiliated Hospital of Sun Yat-sen University, Guangzhou, China

† Both authors have contributed equally to this work.

*Correspondence to: Xiaoming Huang and Ping Han.

We thank Professor Musheng Zeng for his generous help and valuable advice of this study. We have described this in the Acknowledgments part of the current manuscript.

If you agree the changes, please reply this email.

Yours sincerely,

Xiaoming Huang

Re:CDDIS-21-3186RR Author confirmation email

2022-01-06 17:36:03

发件人： "钟茜" <zhongqian@sysucc.org.cn>

收件人： "夏天亮" <xiatl@sysucc.org.cn> "刘尚鑫" <liusx@sysucc.org.cn> "黄晓明" <hxming@mail.sysu.edu.cn> "wangjyi2" <wangjyi2@mail2.sysu.edu.cn> "张华930" <zhangh255@mail.sysu.edu.cn> ... [↓还有6个联系人]

Dear all,

I agree to the changes.

Best,

Qian Zhong

------------------ Original ------------------

From: "夏天亮"<xiatl@sysucc.org.cn>;
Date: Thu, Jan 6, 2022 05:32 PM
To: "刘尚鑫"<liusx@sysucc.org.cn>; "hxming@mail.sysu.edu.cn"<hxming@mail.sysu.edu.cn>; "wangjyi2"<wangjyi2@mail2.sysu.edu.cn>; "钟茜"<zhongqian@sysucc.org.cn>; "张华930"<zhangh255@mail.sysu.edu.cn>; "lishb"<lishb@sysucc.org.cn>; "xiaozhw3"<xiaozhw3@mail.sysu.edu.cn>; "chenrh23"<chenrh23@mail.sysu.edu.cn>; "yeych6"<yeych6@mail2.sysu.edu.cn>; "liangfy3"<liangfy3@mail.sysu.edu.cn>; "hanping5"<hanping5@mail.sysu.edu.cn>;
Subject: Re:CDDIS-21-3186RR Author confirmation email

I agree the changes.

------------------ Original ------------------

From: "刘尚鑫"<liusx@sysucc.org.cn>;
Date: Thu, Jan 6, 2022 05:26 PM
To: "黄晓明"<hxming@mail.sysu.edu.cn>; "wangjyi2"<wangjyi2@mail2.sysu.edu.cn>; "zhongqian"<zhongqian@sysucc.org.cn>; "zhangh255"<zhangh255@mail.sysu.edu.cn>; "lishb"<lishb@sysucc.org.cn>; "夏天亮"<xiatl@sysucc.org.cn>; "xiaozhw3"<xiaozhw3@mail.sysu.edu.cn>; "chenrh23"<chenrh23@mail.sysu.edu.cn>; "yeych6"<yeych6@mail2.sysu.edu.cn>; "liangfy3"<liangfy3@mail.sysu.edu.cn>; "hanping5"<hanping5@mail.sysu.edu.cn>;
Subject: Re:CDDIS-21-3186RR Author confirmation email

I agree the changes.

------------------ Original ------------------

From: "黄晓明"<hxming@mail.sysu.edu.cn>;
Date: Thu, Jan 6, 2022 05:23 PM
To: "hxming"<hxming@mail.sysu.edu.cn>; "wangjyi2"<wangjyi2@mail2.sysu.edu.cn>; "zhongqian"<zhongqian@sysucc.org.cn>; "zhangh255"<zhangh255@mail.sysu.edu.cn>; "刘尚鑫"<liusx@sysucc.org.cn>; "lishb"<lishb@sysucc.org.cn>; "xiatl"<xiatl@sysucc.org.cn>; "xiaozhw3"<xiaozhw3@mail.sysu.edu.cn>; "chenrh23"<chenrh23@mail.sysu.edu.cn>; "yeych6"<yeych6@mail2.sysu.edu.cn>; "liangfy3"<liangfy3@mail.sysu.edu.cn>; "hanping5"<hanping5@mail.sysu.edu.cn>;
Subject: CDDIS-21-3186RR Author confirmation email

Dear co-authors,

According to the contribution work, the co-authors of our manuscript entitled "Nogo-B Promotes Invasion and Metastasis of Nasopharyngeal Carcinoma via RhoA-SRF-MRTFA Pathway" (CDDIS-21-3186R) have been changed as follows:

Jingyi Wang[1†], Qian Zhong[2†], Hua Zhang[3], Shangxin Liu[2], Shibing Li[2], Tianliang Xia[2], Zhiwen Xiao[1,4], Renhui Chen[1], Yuchu Ye[1], Faya Liang[1], Ping Han[1*], Xiaoming Huang[1*]

Re: CDDIS-21-3186RR Author confirmation email

发件人： "张华" <zhangh255@mail.sysu.edu.cn>

收件人： "黄晓明" <hxming@mail.sysu.edu.cn>

2022-01-06 17:32:17

I agree the changes.

-----原始邮件-----
发件人:"黄晓明" <hxming@mail.sysu.edu.cn>
发送时间:2022-01-06 17:22:56 (星期四)
收件人: hxming@mail.sysu.edu.cn, wangjyi2@mail2.sysu.edu.cn, zhongqian@sysucc.org.cn, zhangh255@mail.sysu.edu.cn, liusx@sysucc.org.cn, lishb@sysucc.org.cn, xiatl@sysucc.org.cn, xiaozhw3@mail.sysu.edu.cn, chenrh23@mail.sysu.edu.cn, yeych6@mail2.sysu.edu.cn, liangfy3@mail.sysu.edu.cn, hanping5@mail.sysu.edu.cn
抄送:
主题: CDDIS-21-3186RR Author confirmation email

Dear co-authors,

According to the contribution work, the co-authors of our manuscript entitled "Nogo-B Promotes Invasion and Metastasis of Nasopharyngeal Carcinoma via RhoA-SRF-MRTFA Pathway" (CDDIS-21-3186R) have been changed as follows:

Jingyi Wang[1†], Qian Zhong[2†], Hua Zhang[3], Shangxin Liu[2], Shibing Li[2], Tianliang Xia[2], Zhiwen Xiao[1,4], Renhui Chen[1], Yuchu Ye[1], Faya Liang[1], Ping Han[1*], Xiaoming Huang[1*]

1 Department of Otolaryngology-Head and Neck Surgery, Sun Yat-sen Memorial Hospital, Sun Yat-sen University, Guangzhou, China; Guangdong Provincial Key Laboratory of Malignant Tumor Epigenetics and Gene Regulation, Guangzhou, China

2 State Key Laboratory of Oncology in South China, Guangdong Key Laboratory of Nasopharyngeal Carcinoma Diagnosis and Therapy, Collaborative Innovation Center for Cancer Medicine, Sun Yat-sen University Cancer Center, Guangzhou, China

3 MOE Key Laboratory of Tropical Disease Control, Centre for Infection and Immunity Studies (CIIS), Seventh Affiliated Hospital, School of Medicine, Sun Yat-sen University, Shenzhen, Guangdong, China

4 Department of Otorhinolaryngology Head and Neck Surgery, Department of Thyroid Center/Thyroid Surgery, The Sixth Affiliated Hospital of Sun Yat-sen University, Guangzhou, China

† Both authors have contributed equally to this work.

*Correspondence to: Xiaoming Huang and Ping Han.

We thank Professor Musheng Zeng for his generous help and valuable advice of this study. We have described this in the Acknowledgments part of the current manuscript.

If you agree the changes, please reply this email.

Yours sincerely,

Xiaoming Huang

Jan 6, 2022

Re:CDDIS-21-3186RR Author confirmation email

2022-01-06 17:25:59

发件人： "刘尚鑫" <liusx@sysucc.org.cn>

收件人： "黄晓明" <hxming@mail.sysu.edu.cn> "wangjyi2" <wangjyi2@mail2.sysu.edu.cn> "钟茜" <zhongqian@sysucc.org.cn> "zhangh255" <zhangh255@mail.sysu.edu.cn> "lishb" <lishb@sysucc.org.cn> .. [↓还有6个联系人]

I agree the changes.

------------------ Original ------------------

From: "黄晓明"<hxming@mail.sysu.edu.cn>;
Date: Thu, Jan 6, 2022 05:23 PM
To: "hxming"<hxming@mail.sysu.edu.cn>; "wangjyi2"<wangjyi2@mail2.sysu.edu.cn>; "zhongqian"<zhongqian@sysucc.org.cn>; "zhangh255"<zhangh255@mail.sysu.edu.cn>; "刘尚鑫"<liusx@sysucc.org.cn>; "lishb"<lishb@sysucc.org.cn>; "xiatl"<xiatl@sysucc.org.cn>; "xiaozhw3"<xiaozhw3@mail.sysu.edu.cn>; "chenrh23"<chenrh23@mail.sysu.edu.cn>; "yeych6"<yeych6@mail2.sysu.edu.cn>; "liangfy3"<liangfy3@mail.sysu.edu.cn>; "hanping5"<hanping5@mail.sysu.edu.cn>;
Subject: CDDIS-21-3186RR Author confirmation email

Dear co-authors,

According to the contribution work, the co-authors of our manuscript entitled "Nogo-B Promotes Invasion and Metastasis of Nasopharyngeal Carcinoma via RhoA-SRF-MRTFA Pathway" (CDDIS-21-3186R) have been changed as follows:

Jingyi Wang[1†], Qian Zhong[2†], Hua Zhang[3], Shangxin Liu[2], Shibing Li[2], Tianliang Xia[2], Zhiwen Xiao[1,4], Renhui Chen[1], Yuchu Ye[1], Faya Liang[1], Ping Han[1*], Xiaoming Huang[1*]

1 Department of Otolaryngology-Head and Neck Surgery, Sun Yat-sen Memorial Hospital, Sun Yat-sen University, Guangzhou, China; Guangdong Provincial Key Laboratory of Malignant Tumor Epigenetics and Gene Regulation, Guangzhou, China

2 State Key Laboratory of Oncology in South China, Guangdong Key Laboratory of Nasopharyngeal Carcinoma Diagnosis and Therapy, Collaborative Innovation Center for Cancer Medicine, Sun Yat-sen University Cancer Center, Guangzhou, China

3 MOE Key Laboratory of Tropical Disease Control, Centre for Infection and Immunity Studies (CIIS), Seventh Affiliated Hospital, School of Medicine, Sun Yat-sen University, Shenzhen, Guangdong, China

4 Department of Otorhinolaryngology Head and Neck Surgery, Department of Thyroid Center/Thyroid Surgery, The Sixth Affiliated Hospital of Sun Yat-sen University, Guangzhou, China

[†] Both authors have contributed equally to this work.

*Correspondence to: Xiaoming Huang and Ping Han.

We thank Professor Musheng Zeng for his generous help and valuable advice of this study. We have described this in the Acknowledgments part of the current manuscript.

If you agree the changes, please reply this email.

Yours sincerely,

Xiaoming Huang

Jan 6, 2022

Re:CDDIS-21-3186RR Author confirmation email

2022-01-06 18:20:58

发件人： "李世兵" <lishb@sysucc.org.cn>

收件人： "黄晓明" <hxming@mail.sysu.edu.cn> "wangjyi2" <wangjyi2@mail2.sysu.edu.cn> "钟茜" <zhongqian@sysucc.org.cn> "zhangh255" <zhangh255@mail.sysu.edu.cn> "liusx" <liusx@sysucc.org.cn> - [↓还有6个联系人]

I agree the changes

Shibing Li

------------------ Original ------------------

From: "黄晓明"<hxming@mail.sysu.edu.cn>;
Date: Thu, Jan 6, 2022 05:23 PM
To: "hxming"<hxming@mail.sysu.edu.cn>; "wangjyi2"<wangjyi2@mail2.sysu.edu.cn>; "zhongqian"<zhongqian@sysucc.org.cn>; "zhangh255"<zhangh255@mail.sysu.edu.cn>; "liusx"<liusx@sysucc.org.cn>; "李世兵"<lishb@sysucc.org.cn>; "xiatl"<xiatl@sysucc.org.cn>; "xiaozhw3"<xiaozhw3@mail.sysu.edu.cn>; "chenrh23"<chenrh23@mail.sysu.edu.cn>; "yeych6"<yeych6@mail2.sysu.edu.cn>; "liangfy3"<liangfy3@mail.sysu.edu.cn>; "hanping5"<hanping5@mail.sysu.edu.cn>;
Subject: CDDIS-21-3186RR Author confirmation email

Dear co-authors,

According to the contribution work, the co-authors of our manuscript entitled "Nogo-B Promotes Invasion and Metastasis of Nasopharyngeal Carcinoma via RhoA-SRF-MRTFA Pathway" (CDDIS-21-3186R) have been changed as follows:

Jingyi Wang[1†], Qian Zhong[2†], Hua Zhang[3], Shangxin Liu[2], Shibing Li[2], Tianliang Xia[2], Zhiwen Xiao[1,4], Renhui Chen[1], Yuchu Ye[1], Faya Liang[1], Ping Han[1*], Xiaoming Huang[1*]

1 Department of Otolaryngology-Head and Neck Surgery, Sun Yat-sen Memorial Hospital, Sun Yat-sen University, Guangzhou, China; Guangdong Provincial Key Laboratory of Malignant Tumor Epigenetics and Gene Regulation, Guangzhou, China

2 State Key Laboratory of Oncology in South China, Guangdong Key Laboratory of Nasopharyngeal Carcinoma Diagnosis and Therapy, Collaborative Innovation Center for Cancer Medicine, Sun Yat-sen University Cancer Center, Guangzhou, China

3 MOE Key Laboratory of Tropical Disease Control, Centre for Infection and Immunity Studies (CIIS), Seventh Affiliated Hospital, School of Medicine, Sun Yat-sen University, Shenzhen, Guangdong, China

4 Department of Otorhinolaryngology Head and Neck Surgery, Department of Thyroid Center/Thyroid Surgery, The Sixth Affiliated Hospital of Sun Yat-sen University, Guangzhou, China

† Both authors have contributed equally to this work.

*Correspondence to: Xiaoming Huang and Ping Han.

We thank Professor Musheng Zeng for his generous help and valuable advice of this study. We have described this in the Acknowledgments part of the current manuscript.

If you agree the changes, please reply this email.

Yours sincerely,

Xiaoming Huang

Jan 6, 2022

快捷回复给所有人

Re:CDDIS-21-3186RR Author confirmation email

发件人： "夏天亮" <xiatl@sysucc.org.cn>

收件人： "刘尚鑫" <liusx@sysucc.org.cn> "黄晓明" <hxming@mail.sysu.edu.cn> "wangjyi2" <wangjyi2@mail2.sysu.edu.cn> "钟茜" <zhongqian@sysucc.org.cn> "zhangh255" <zhangh255@mail.sysu.edu.cn> ... [还有6个联系人]

2022-01-06 17:32:38

I agree the changes.

------------------ Original ------------------

From: "刘尚鑫"<liusx@sysucc.org.cn>;
Date: Thu, Jan 6, 2022 05:26 PM
To: "黄晓明"<hxming@mail.sysu.edu.cn>; "wangjyi2"<wangjyi2@mail2.sysu.edu.cn>; "zhongqian"<zhongqian@sysucc.org.cn>; "zhangh255"<zhangh255@mail.sysu.edu.cn>; "lishb"<lishb@sysucc.org.cn>; "夏天亮"<xiatl@sysucc.org.cn>; "xiaozhw3"<xiaozhw3@mail.sysu.edu.cn>; "chenrh23"<chenrh23@mail.sysu.edu.cn>; "yeych6"<yeych6@mail2.sysu.edu.cn>; "liangfy3"<liangfy3@mail.sysu.edu.cn>; "hanping5"<hanping5@mail.sysu.edu.cn>;
Subject: Re:CDDIS-21-3186RR Author confirmation email

I agree the changes.

------------------ Original ------------------

From: "黄晓明"<hxming@mail.sysu.edu.cn>;
Date: Thu, Jan 6, 2022 05:23 PM
To: "hxming"<hxming@mail.sysu.edu.cn>; "wangjyi2"<wangjyi2@mail2.sysu.edu.cn>; "zhongqian"<zhongqian@sysucc.org.cn>; "zhangh255"<zhangh255@mail.sysu.edu.cn>; "刘尚鑫"<liusx@sysucc.org.cn>; "lishb"<lishb@sysucc.org.cn>; "xiatl"<xiatl@sysucc.org.cn>; "xiaozhw3"<xiaozhw3@mail.sysu.edu.cn>; "chenrh23"<chenrh23@mail.sysu.edu.cn>; "yeych6"<yeych6@mail2.sysu.edu.cn>; "liangfy3"<liangfy3@mail.sysu.edu.cn>; "hanping5"<hanping5@mail.sysu.edu.cn>;
Subject: CDDIS-21-3186RR Author confirmation email

Dear co-authors,

According to the contribution work, the co-authors of our manuscript entitled "Nogo-B Promotes Invasion and Metastasis of Nasopharyngeal Carcinoma via RhoA-SRF-MRTFA Pathway" (CDDIS-21-3186R) have been changed as follows:

Jingyi Wang[1†], Qian Zhong[2†], Hua Zhang[3], Shangxin Liu[2], Shibing Li[2], Tianliang Xia[2], Zhiwen Xiao[1,4], Renhui Chen[1], Yuchu Ye[1], Faya Liang[1], Ping Han[1*], Xiaoming Huang[1*]

1 Department of Otolaryngology-Head and Neck Surgery, Sun Yat-sen Memorial Hospital, Sun Yat-sen University, Guangzhou, China; Guangdong Provincial Key Laboratory of Malignant Tumor Epigenetics and Gene Regulation, Guangzhou, China

2 State Key Laboratory of Oncology in South China, Guangdong Key Laboratory of Nasopharyngeal Carcinoma Diagnosis and Therapy, Collaborative Innovation Center for Cancer Medicine, Sun Yat-sen University Cancer Center, Guangzhou, China

3 MOE Key Laboratory of Tropical Disease Control, Centre for Infection and Immunity Studies (CIIS), Seventh Affiliated Hospital, School of Medicine, Sun Yat-sen University, Shenzhen, Guangdong, China

4 Department of Otorhinolaryngology Head and Neck Surgery, Department of Thyroid Center/Thyroid Surgery, The Sixth Affiliated Hospital of Sun Yat-sen University, Guangzhou, China

[†] Both authors have contributed equally to this work.

*Correspondence to: Xiaoming Huang and Ping Han.

We thank Professor Musheng Zeng for his generous help and valuable advice of this study. We have described this in the Acknowledgments part of the current manuscript.

If you agree the changes, please reply this email.

快捷回复给所有人

Re: Re:CDDIS-21-3186RR Author confirmation email

发件人： "肖志文" <xiaozhw3@mail.sysu.edu.cn>

收件人： "夏天亮" <xiatl@sysucc.org.cn>

抄 送： "刘尚鑫" <liusx@sysucc.org.cn> "黄晓明" <hxming@mail.sysu.edu.cn> "wangjyi2" <wangjyi2@mail2.sysu.edu.cn> "钟茜" <zhongqian@sysucc.org.cn> "zhangh255" <zhangh255@mail.sysu.edu.cn> .. [↓还有5个联系人]

2022-01-06 18:02:53

I agree the changes.

Zhiwen Xiao

肖志文

------原始邮件------
发件人:"夏天亮" <xiatl@sysucc.org.cn>
发送时间:2022-01-06 17:32:38 (星期四)
收件人: "刘尚鑫" <liusx@sysucc.org.cn>, "黄晓明" <hxming@mail.sysu.edu.cn>, wangjyi2 <wangjyi2@mail2.sysu.edu.cn>, zhongqian <zhongqian@sysucc.org.cn>, zhangh255 <zhangh255@mail.sysu.edu.cn>, lishb <lishb@sysucc.org.cn>, xiaozhw3 <xiaozhw3@mail.sysu.edu.cn>, chenrh23 <chenrh23@mail.sysu.edu.cn>, yeych6 <yeych6@mail2.sysu.edu.cn>, liangfy3 <liangfy3@mail.sysu.edu.cn>, hanping5 <hanping5@mail.sysu.edu.cn>
抄送:
主题: Re:CDDIS-21-3186RR Author confirmation email

I agree the changes.

------------------ Original ------------------

From: "刘尚鑫"<liusx@sysucc.org.cn>;
Date: Thu, Jan 6, 2022 05:26 PM
To: "黄晓明"<hxming@mail.sysu.edu.cn>; "wangjyi2"<wangjyi2@mail2.sysu.edu.cn>; "zhongqian"<zhongqian@sysucc.org.cn>; "zhangh255"<zhangh255@mail.sysu.edu.cn>; "lishb"<lishb@sysucc.org.cn>; "夏天亮"<xiatl@sysucc.org.cn>; "xiaozhw3"<xiaozhw3@mail.sysu.edu.cn>; "chenrh23"<chenrh23@mail.sysu.edu.cn>; "yeych6"<yeych6@mail2.sysu.edu.cn>; "liangfy3"<liangfy3@mail.sysu.edu.cn>; "hanping5"<hanping5@mail.sysu.edu.cn>;
Subject: Re:CDDIS-21-3186RR Author confirmation email

I agree the changes.

------------------ Original ------------------

From: "黄晓明"<hxming@mail.sysu.edu.cn>;
Date: Thu, Jan 6, 2022 05:23 PM
To: "hxming"<hxming@mail.sysu.edu.cn>; "wangjyi2"<wangjyi2@mail2.sysu.edu.cn>; "zhongqian"<zhongqian@sysucc.org.cn>; "zhangh255"<zhangh255@mail.sysu.edu.cn>; "刘尚鑫"<liusx@sysucc.org.cn>; "lishb"<lishb@sysucc.org.cn>; "xiatl"<xiatl@sysucc.org.cn>; "xiaozhw3"<xiaozhw3@mail.sysu.edu.cn>; "chenrh23"<chenrh23@mail.sysu.edu.cn>; "yeych6"<yeych6@mail2.sysu.edu.cn>; "liangfy3"<liangfy3@mail.sysu.edu.cn>; "hanping5"<hanping5@mail.sysu.edu.cn>;
Subject: CDDIS-21-3186RR Author confirmation email

Dear co-authors,

[illegible]

Re: CDDIS-21-3186RR Author confirmation email

发件人： "陈仁晖" <chenrh23@mail.sysu.edu.cn>

收件人： "黄晓明" <hxming@mail.sysu.edu.cn>

抄 送： wangjyi2@mail2.sysu.edu.cn "钟茜" <zhongqian@sysucc.org.cn> zhangh255@mail.sysu.edu.cn liusx@sysucc.org.cn lishb@sysucc.org.cn - [↓还有5个联系人]

2022-01-07 11:15:01

Dear all,

I agree to the changes.

Best,

Renhui Chen

2022-01-06 17:22:56"黄晓明" <hxming@mail.sysu.edu.cn>写道：

> Dear co-authors,
>
> According to the contribution work, the co-authors of our manuscript entitled "Nogo-B Promotes Invasion and Metastasis of Nasopharyngeal Carcinoma via RhoA-SRF-MRTFA Pathway" (CDDIS-21-3186R) have been changed as follows:
>
> Jingyi Wang[1†], Qian Zhong[2†], Hua Zhang[3], Shangxin Liu[2], Shibing Li[2], Tianliang Xia[2], Zhiwen Xiao[1,4], Renhui Chen[1], Yuchu Ye[1], Faya Liang[1], Ping Han[1*], Xiaoming Huang[1*]
>
> 1 Department of Otolaryngology-Head and Neck Surgery, Sun Yat-sen Memorial Hospital, Sun Yat-sen University, Guangzhou, China; Guangdong Provincial Key Laboratory of Malignant Tumor Epigenetics and Gene Regulation, Guangzhou, China
>
> 2 State Key Laboratory of Oncology in South China, Guangdong Key Laboratory of Nasopharyngeal Carcinoma Diagnosis and Therapy, Collaborative Innovation Center for Cancer Medicine, Sun Yat-sen University Cancer Center, Guangzhou, China
>
> 3 MOE Key Laboratory of Tropical Disease Control, Centre for Infection and Immunity Studies (CIIS), Seventh Affiliated Hospital, School of Medicine, Sun Yat-sen University, Shenzhen, Guangdong, China
>
> 4 Department of Otorhinolaryngology Head and Neck Surgery, Department of Thyroid Center/Thyroid Surgery, The Sixth Affiliated Hospital of Sun Yat-sen University, Guangzhou, China
>
> † Both authors have contributed equally to this work.
>
> *Correspondence to: Xiaoming Huang and Ping Han.
>
> We thank Professor Musheng Zeng for his generous help and valuable advice of this study. We have described this in the Acknowledgments part of the current manuscript.
>
> If you agree the changes, please reply this email.
>
> Yours sincerely,
>
> Xiaoming Huang
>
> Jan 6, 2022

Re:CDDIS-21-3186RR Author confirmation email

发件人： "叶煜初" <yeych6@mail2.sysu.edu.cn>

收件人： "黄晓明" <hxming@mail.sysu.edu.cn> "wangjyi2" <wangjyi2@mail2.sysu.edu.cn> "钟茜" <zhongqian@sysucc.org.cn> "zhangh255" <zhangh255@mail.sysu.edu.cn> "liusx" <liusx@sysucc.org.cn> - [↓还有6个联系人]

2022-01-07 11:16:15

I agree to the changes
Yuchu Ye

------------------ Original ------------------

From: "黄晓明"<hxming@mail.sysu.edu.cn>;
Date: Thu, Jan 6, 2022 05:23 PM
To: "hxming"<hxming@mail.sysu.edu.cn>; "wangjyi2"<wangjyi2@mail2.sysu.edu.cn>; "zhongqian"<zhongqian@sysucc.org.cn>; "zhangh255"<zhangh255@mail.sysu.edu.cn>; "liusx"<liusx@sysucc.org.cn>; "lishb"<lishb@sysucc.org.cn>; "xiatl"<xiatl@sysucc.org.cn>; "xiaozhw3"<xiaozhw3@mail.sysu.edu.cn>; "chenrh23"<chenrh23@mail.sysu.edu.cn>; "叶煜初"<yeych6@mail2.sysu.edu.cn>; "liangfy3"<liangfy3@mail.sysu.edu.cn>; "hanping5"<hanping5@mail.sysu.edu.cn>;
Subject: CDDIS-21-3186RR Author confirmation email

Dear co-authors,

According to the contribution work, the co-authors of our manuscript entitled "Nogo-B Promotes Invasion and Metastasis of Nasopharyngeal Carcinoma via RhoA-SRF-MRTFA Pathway" (CDDIS-21-3186R) have been changed as follows:

Jingyi Wang[1†], Qian Zhong[2†], Hua Zhang[3], Shangxin Liu[2], Shibing Li[2], Tianliang Xia[2], Zhiwen Xiao[1,4], Renhui Chen[1], Yuchu Ye[1], Faya Liang[1], Ping Han[1*], Xiaoming Huang[1*]

1 Department of Otolaryngology-Head and Neck Surgery, Sun Yat-sen Memorial Hospital, Sun Yat-sen University, Guangzhou, China; Guangdong Provincial Key Laboratory of Malignant Tumor Epigenetics and Gene Regulation, Guangzhou, China

2 State Key Laboratory of Oncology in South China, Guangdong Key Laboratory of Nasopharyngeal Carcinoma Diagnosis and Therapy, Collaborative Innovation Center for Cancer Medicine, Sun Yat-sen University Cancer Center, Guangzhou, China

3 MOE Key Laboratory of Tropical Disease Control, Centre for Infection and Immunity Studies (CIIS), Seventh Affiliated Hospital, School of Medicine, Sun Yat-sen University, Shenzhen, Guangdong, China

4 Department of Otorhinolaryngology Head and Neck Surgery, Department of Thyroid Center/Thyroid Surgery, The Sixth Affiliated Hospital of Sun Yat-sen University, Guangzhou, China

[†] Both authors have contributed equally to this work.

*Correspondence to: Xiaoming Huang and Ping Han.

We thank Professor Musheng Zeng for his generous help and valuable advice of this study. We have described this in the Acknowledgments part of the current manuscript.

If you agree the changes, please reply this email.

Yours sincerely,

Xiaoming Huang

Jan 6, 2022

Re: CDDIS-21-3186RR Author confirmation email

发件人： "liangfy3" <liangfy3@mail.sysu.edu.cn>

收件人： "黄晓明" <hxming@mail.sysu.edu.cn>

2022-01-06 17:31:35

I agree the changes.

Faya Liang

发自我的iPhone

------------------ Original ------------------

From: 黄晓明 <hxming@mail.sysu.edu.cn>
Date: Thu, Jan 6, 2022 5:22 PM
To: hxming <hxming@mail.sysu.edu.cn>, wangjyi2 <wangjyi2@mail2.sysu.edu.cn>, zhongqian <zhongqian@sysucc.org.cn>, zhangh255 <zhangh255@mail.sysu.edu.cn>, liusx <liusx@sysucc.org.cn>, lishb <lishb@sysucc.org.cn>, xiatl <xiatl@sysucc.org.cn>, xiaozhw3 <xiaozhw3@mail.sysu.edu.cn>, chenrh23 <chenrh23@mail.sysu.edu.cn>, yeych6 <yeych6@mail2.sysu.edu.cn>, liangfy3 <liangfy3@mail.sysu.edu.cn>, hanping5 <hanping5@mail.sysu.edu.cn>
Subject: Re: CDDIS-21-3186RR Author confirmation email

Dear co-authors,

According to the contribution work, the co-authors of our manuscript entitled “Nogo-B Promotes Invasion and Metastasis of Nasopharyngeal Carcinoma via RhoA-SRF-MRTFA Pathway” (CDDIS-21-3186R) have been changed as follows:

Jingyi Wang[1†], Qian Zhong[2†], Hua Zhang[3], Shangxin Liu[2], Shibing Li[2], Tianliang Xia[2], Zhiwen Xiao[1,4], Renhui Chen[1], Yuchu Ye[1], Faya Liang[1], Ping Han[1*], Xiaoming Huang[1*]

1 Department of Otolaryngology-Head and Neck Surgery, Sun Yat-sen Memorial Hospital, Sun Yat-sen University, Guangzhou, China; Guangdong Provincial Key Laboratory of Malignant Tumor Epigenetics and Gene Regulation, Guangzhou, China

2 State Key Laboratory of Oncology in South China, Guangdong Key Laboratory of Nasopharyngeal Carcinoma Diagnosis and Therapy, Collaborative Innovation Center for Cancer Medicine, Sun Yat-sen University Cancer Center, Guangzhou, China

3 MOE Key Laboratory of Tropical Disease Control, Centre for Infection and Immunity Studies (CIIS), Seventh Affiliated Hospital, School of Medicine, Sun Yat-sen University, Shenzhen, Guangdong, China

4 Department of Otorhinolaryngology Head and Neck Surgery, Department of Thyroid Center/Thyroid Surgery, The Sixth Affiliated Hospital of Sun Yat-sen University, Guangzhou, China

[†] Both authors have contributed equally to this work.

*Correspondence to: Xiaoming Huang and Ping Han.

We thank Professor Musheng Zeng for his generous help and valuable advice of this study. We have described this in the Acknowledgments part of the current manuscript.

If you agree the changes, please reply this email.

Yours sincerely,

[illegible]

Re: CDDIS-21-3186RR Author confirmation email

2022-01-06 17:26:59

发件人： "hanping5@mail.sysu.edu.cn" <hanping5@mail.sysu.edu.cn>

收件人： "黄晓明" <hxming@mail.sysu.edu.cn>

Yes, I agree.

Ping Han
Jan 6, 2022

---Original---

From: "黄晓明"<hxming@mail.sysu.edu.cn>
Date: Thu, Jan 6, 2022 17:22 PM
To: "hxming"<hxming@mail.sysu.edu.cn>;"wangjyi2"<wangjyi2@mail2.sysu.edu.cn>;"zhongqian"<zhongqian@sysucc.org.cn>;"zhangh255"<zhangh255@mail.sysu.edu.cn>;"liusx"<liusx@sysucc.org.cn>;"lishb"<lishb@sysucc.org.cn>;"xiatl"<xiatl@sysucc.org.cn>;"xiaozhw3"<xiaozhw3@mail.sysu.edu.cn>;"chenrh23"<chenrh23@mail.sysu.edu.cn>;"yeych6"<yeych6@mail2.sysu.edu.cn>;"liangfy3"<liangfy3@mail.sysu.edu.cn>;"hanping5"<hanping5@mail.sysu.edu.cn>;
Subject: CDDIS-21-3186RR Author confirmation email

Dear co-authors,

According to the contribution work, the co-authors of our manuscript entitled "Nogo-B Promotes Invasion and Metastasis of Nasopharyngeal Carcinoma via RhoA-SRF-MRTFA Pathway" (CDDIS-21-3186R) have been changed as follows:

Jingyi Wang[1†], Qian Zhong[2†], Hua Zhang[3], Shangxin Liu[2], Shibing Li[2], Tianliang Xia[2], Zhiwen Xiao[1,4], Renhui Chen[1], Yuchu Ye[1], Faya Liang[1], Ping Han[1*], Xiaoming Huang[1*]

1 Department of Otolaryngology-Head and Neck Surgery, Sun Yat-sen Memorial Hospital, Sun Yat-sen University, Guangzhou, China; Guangdong Provincial Key Laboratory of Malignant Tumor Epigenetics and Gene Regulation, Guangzhou, China

2 State Key Laboratory of Oncology in South China, Guangdong Key Laboratory of Nasopharyngeal Carcinoma Diagnosis and Therapy, Collaborative Innovation Center for Cancer Medicine, Sun Yat-sen University Cancer Center, Guangzhou, China

3 MOE Key Laboratory of Tropical Disease Control, Centre for Infection and Immunity Studies (CIIS), Seventh Affiliated Hospital, School of Medicine, Sun Yat-sen University, Shenzhen, Guangdong, China

4 Department of Otorhinolaryngology Head and Neck Surgery, Department of Thyroid Center/Thyroid Surgery, The Sixth Affiliated Hospital of Sun Yat-sen University, Guangzhou, China

[†] Both authors have contributed equally to this work.

*Correspondence to: Xiaoming Huang and Ping Han.

We thank Professor Musheng Zeng for his generous help and valuable advice of this study. We have described this in the Acknowledgments part of the current manuscript.

If you agree the changes, please reply this email.

Yours sincerely,

Xiaoming Huang

Jan 6, 2022

Re: CDDIS-21-3186RR Author confirmation email

2022-01-06 17:46:57

发件人： "黄晓明" <hxming@mail.sysu.edu.cn>

收件人： wangjyi2@mail2.sysu.edu.cn "钟茜" <zhongqian@sysucc.org.cn> zhangh255@mail.sysu.edu.cn liusx@sysucc.org.cn lishb@sysucc.org.cn - [↓还有6个联系人]

对方已阅读 查看详情 共发给11个收件人，其中 6个成功到达对方服务器，4个信件已被对方阅读，1个成功到达对方邮箱

Dear all,

I agree to the changes.

Yours sincerely,

Xiaoming Huang

2022-01-06 17:22:56"黄晓明" <hxming@mail.sysu.edu.cn>写道：

Dear co-authors,

According to the contribution work, the co-authors of our manuscript entitled "Nogo-B Promotes Invasion and Metastasis of Nasopharyngeal Carcinoma via RhoA-SRF-MRTFA Pathway" (CDDIS-21-3186R) have been changed as follows:

Jingyi Wang[1†], Qian Zhong[2†], Hua Zhang[3], Shangxin Liu[2], Shibing Li[2], Tianliang Xia[2], Zhiwen Xiao[1,4], Renhui Chen[1], Yuchu Ye[1], Faya Liang[1], Ping Han[1*], Xiaoming Huang[1*]

1 Department of Otolaryngology-Head and Neck Surgery, Sun Yat-sen Memorial Hospital, Sun Yat-sen University, Guangzhou, China; Guangdong Provincial Key Laboratory of Malignant Tumor Epigenetics and Gene Regulation, Guangzhou, China

2 State Key Laboratory of Oncology in South China, Guangdong Key Laboratory of Nasopharyngeal Carcinoma Diagnosis and Therapy, Collaborative Innovation Center for Cancer Medicine, Sun Yat-sen University Cancer Center, Guangzhou, China

3 MOE Key Laboratory of Tropical Disease Control, Centre for Infection and Immunity Studies (CIIS), Seventh Affiliated Hospital, School of Medicine, Sun Yat-sen University, Shenzhen, Guangdong, China

4 Department of Otorhinolaryngology Head and Neck Surgery, Department of Thyroid Center/Thyroid Surgery, The Sixth Affiliated Hospital of Sun Yat-sen University, Guangzhou, China

† Both authors have contributed equally to this work.

*Correspondence to: Xiaoming Huang and Ping Han.

We thank Professor Musheng Zeng for his generous help and valuable advice of this study. We have described this in the Acknowledgments part of the current manuscript.

If you agree the changes, please reply this email.

Yours sincerely,

Xiaoming Huang

Jan 6, 2022
